# Bio-organic fertilizers stimulate indigenous soil *Pseudomonas* populations to enhance plant disease suppression

**DOI:** 10.1186/s40168-020-00892-z

**Published:** 2020-09-22

**Authors:** Chengyuan Tao, Rong Li, Wu Xiong, Zongzhuan Shen, Shanshan Liu, Beibei Wang, Yunze Ruan, Stefan Geisen, Qirong Shen, George A. Kowalchuk

**Affiliations:** 1 Jiangsu Provincial Key Lab of Solid Organic Waste Utilization, Jiangsu Collaborative Innovation Center of Solid Organic Wastes, Educational Ministry Engineering Center of Resource-saving fertilizers, Nanjing Agricultural University, Nanjing, 210095 Jiangsu People’s Republic of China; 2 The Key Laboratory of Plant Immunity, Nanjing Agricultural University, Nanjing, 210095 Jiangsu People’s Republic of China; 3 Ecology and Biodiversity Group, Department of Biology, Institute of Environmental Biology, Utrecht University, 3584 Utrecht, CH Netherlands; 4 Hainan key Laboratory for Sustainable Utilization of Tropical Bio-resources, College of tropical crops, Hainan University, Haikou, 570228 People’s Republic of China; 5 Department of Terrestrial Ecology, Netherlands Institute for Ecology, (NIOO-KNAW), 6708 Wageningen, PB Netherlands; 6 Laboratory of Nematology, Wageningen University, 6700 Wageningen, AA Netherlands; 7 College of Resources and Environmental Sciences, Nanjing Agricultural University, Nanjing, 210095 People’s Republic of China

**Keywords:** Bio-organic fertilizer, Fusarium wilt, Disease suppression, Resident microbiota, *Pseudomonas* spp., Interspecific synergy

## Abstract

**Background:**

Plant diseases caused by fungal pathogen result in a substantial economic impact on the global food and fruit industry. Application of organic fertilizers supplemented with biocontrol microorganisms (*i.e.* bioorganic fertilizers) has been shown to improve resistance against plant pathogens at least in part due to impacts on the structure and function of the resident soil microbiome. However, it remains unclear whether such improvements are driven by the specific action of microbial inoculants, microbial populations naturally resident to the organic fertilizer or the physical-chemical properties of the compost substrate. The aim of this study was to seek the ecological mechanisms involved in the disease suppressive activity of bio-organic fertilizers.

**Results:**

To disentangle the mechanism of bio-organic fertilizer action, we conducted an experiment tracking Fusarium wilt disease of banana and changes in soil microbial communities over three growth seasons in response to the following four treatments: bio-organic fertilizer (containing *Bacillus amyloliquefaciens* W19), organic fertilizer, sterilized organic fertilizer and sterilized organic fertilizer supplemented with *B*. *amyloliquefaciens* W19. We found that sterilized bioorganic fertilizer to which *Bacillus* was re-inoculated provided a similar degree of disease suppression as the non-sterilized bioorganic fertilizer across cropping seasons. We further observed that disease suppression in these treatments is linked to impacts on the resident soil microbial communities, specifically by leading to increases in specific *Pseudomonas* spp.. Observed correlations between *Bacillus* amendment and indigenous *Pseudomonas* spp. that might underlie pathogen suppression were further studied in laboratory and pot experiments. These studies revealed that specific bacterial taxa synergistically increase biofilm formation and likely acted as a plant-beneficial consortium against the pathogen.

**Conclusion:**

Together we demonstrate that the action of bioorganic fertilizer is a product of the biocontrol inoculum within the organic amendment and its impact on the resident soil microbiome. This knowledge should help in the design of more efficient biofertilizers designed to promote soil function.

Video Abstract

## Background

Soils are critical to human wellbeing by providing food, feed, fiber, and medicine [1]. Soil organisms are pivotal agents in supplying these ecosystem services [2], for instance by driving nutrient cycling, transformation of organic materials, enhancing plant productivity and helping to control against soil-borne diseases [3–5]. At a systems level, the microbiome plays an integral role in virtually all soil processes, such that microbial abundance, composition and activity will largely determine sustainable productivity of agricultural land [4, 6, 7]. The soil microbiome can be influenced either positively or negatively by soil management or perturbations, resulting in taxonomic and functional changes in the soil microbiome [7, 8]. As such, identifying factors that affect the soil microbiome is a prerequisite to the development of targeted manipulations to increase soil service provisioning [2, 9–11].

A variety of soil-borne diseases are increasingly threatening stable agricultural production around the world [12, 13]. Soil-borne microbes can play an important role in limiting the damage inflicted by such diseases [13–15]. For instance, a range of disease-suppressive soils has been described in which either specific components or general community action contributes to resistance against soil-borne fungal pathogens [16–19]. As a logical extension of such findings, introduction of microbes contributing to disease suppression holds promise as a sustainable strategy for the control of plant disease [1, 9, 10]. Soil-borne microbial diversity is vast, with a wide range of plant-microbe interactions spanning the scale from highly beneficial to neutral to deleterious for the plant [13, 14, 18]. In this light, the ability to manage the soil microbiome to increase the abundance of beneficial and reduce detrimental interactions holds a large potential for the development of more sustainable agricultural systems [9, 16, 20, 21]. However, optimizing soil-microbe-plant partnerships to increase soil functionality against pathogens is a daunting task given the complexity of plant-microorganism and microorganism-microorganism interactions [9, 11, 22].

The use of plant probiotic microorganisms has been shown to hold promise for improving plant health, nutrition, and stress resilience [18, 23–25], and the delivery of such plant probiotics via for instance bioorganic fertilizers has proven particularly effective in improving soil microbial functionality [26–29]. Although potentially effective, the mechanisms driving the success of such bioorganic fertilizer applications are generally not well described. Multiple modes of action are possible, including direct antagonism of the pathogen [30–33], induction of systemic resistance in plants (ISR) [33–35] or indirect impacts on the pathogen via effects on the resident soil microbiome [36, 37]. Within the soil microbiome, previous studies have shown that specific microbial groups related to plant disease suppression (such as *Pseudomonas*, *Streptomyces*, *Flavobacterium*, etc.) [17, 19, 38] may be stimulated by bioorganic fertilizer applications [36]. Thus, strategies that stimulate the activities of these soil-borne microbial groups may be particularly effective in helping to suppress plant diseases. In addition, little is known about which components of bioorganic fertilizers are most responsible for yielding disease-suppressive effects upon application. These components include the biocontrol agent itself, the physical-chemical nature of the compost substrate and the microbial community resident to the compost. Disentangling the relative importance of specific components of bioorganic fertilizers and understanding their mode of action is an important step toward designing and optimizing strategies for the effective enhancement of soil microbiome functioning.

In this study, we carried out an experiment focused on continuous cropping of banana in a soil infested with Fusarium wilt disease. Four treatments were carried out with addition of either sterilized or non-sterilized organic fertilizer each either inoculated with a biocontrol strain (*B*. *amyloliquefaciens* W19) or receiving no inoculum. This design allowed us to disentangle the relative contribution of the organic substrate addition, the fertilizer microbiome and the inoculated biocontrol strain on disease suppression. We tracked the soil microbial communities across treatments to examine the potential role of changes in resident soil communities in disease suppression. Additional analyses zoomed in on the genus *Pseudomonas* as a soil-borne microbial group with demonstrated impacts on disease suppression [17, 33, 35]. By determining the mode of action of bioorganic fertilizers, we sought to provide the necessary understanding required for the more efficient and informed development of soil microbiome manipulation strategies involving biologically enhanced organic fertilizers.

## Methods

### Experimental design

We established a series of mesocosms for banana cultivation in a greenhouse located at the WanZhong Co., Ltd. in Jianfeng town, Ledong County, Hainan Province, China (108°45′E, 18°38′N). Mesocosms were constructed from polypropylene pots (25 × 30 × 30 cm) filled with 10 kg soil. The soil was loam sandy dry red soil collected from a field with a history of more than 10 years of banana monoculture cultivation and a high level of Fusarium wilt disease (approximately 60% at the time of soil collection). The soil had a pH of 5.75, a total C content of 4.42 g/kg, a total N content of 0.63 g/kg, and available P, K contents of 68.88, 360.33 mg/kg, respectively. Four different fertilizer treatments were applied as follows: OF, soil amended with organic fertilizer; OF+W19, soil amended with bio-organic fertilizer containing *B*. *amyloliquefaciens* W19; SOF, soil amended with sterilized organic fertilizer; and SOF+W19, soil amended with sterilized organic fertilizer supplemented with *B*. *amyloliquefaciens* W19. The mesocosm experiment was performed using a randomized complete block design with three replicates for each treatment, and each replicate contained ten pots. Each pot received one banana seedling (*Musa* AAA Cavendish cv. Brazil), which was provided by Hainan Wan Zhong Co., Ltd [39]. Bio-organic and organic fertilizers were produced as described by Wang [39]. Fertilizer sterilization was performed by Co75 γ-ray irradiation at Nanjing Xiyue Technology Co., Ltd, Nanjing, China. The population density of strain W19 in the SOF+W19 treatment was confirmed to be at least 1.0×10^9^ CFU g^-1^ dry weight of fertilizer at the start of the experiment. Each pot was supplemented with 180 g of the given amendment before banana seedlings were transplanted for each of three successive seasons, with each successive season using soil from the previous year after plant removal. Incidence of Fusarium wilt disease was monitored as described by Jeger [40] and calculated as the percentage of infected plants relative to the total number of plants.

### Soil sampling and DNA extraction

Bulk and rhizosphere soil samples were collected 4 months after seedling transplantation for each season of the greenhouse experiment. Bulk soil samples were collected by first removing banana plants and then taking soil cores to a depth of 10 cm. Representative bulk soil samples were obtained by combining the samples from three pots in a given replicate and subsequent passage through a 2 mm sieve [36]. Sampling of rhizosphere soil was performed as described by Fu [37]. Briefly, soil tightly bound to the roots was recovered by rinsing with sterile saline solution, and this soil suspension was centrifuged at 10 000 x *g* for 10 min, with the resulting pellet defined as rhizosphere soil. All bulk and rhizosphere soil samples were stored at -80^o^C prior to DNA extraction, and for each soil sample (24 in total: 4 treatments × 3 replicates × 2 positions (bulk and rhizosphere)), total soil genomic DNA was extracted from 0.5 g soil using the PowerSoil DNA Isolation Kit (Mobio Laboratories, Carlsbad, CA, USA) following the manufacturer's instructions. The concentration and quality of the DNA was determined using a NanoDrop 2000 spectrophotometer (Thermo Scientific, Waltham, MA, USA).

### Tag sequencing for bacterial and fungal communities analysis

Bacterial and fungal sequencing libraries were constructed according to previously described protocols [41, 42]. Investigation of bacterial and fungal communities was based on paired-end amplicon sequencing of the 16S rRNA gene and the ITS region of fungal ribosomal DNA on an Illumina MiSeq PE 250 platform at Personal Biotechnology Co., Ltd (Shanghai, China). Amplification of bacterial 16S rRNA gene fragments was performed using the general bacterial primers 520F (5’-AYT GGG YDT AAA GNG-3’) and 802R (5’-TAC NVG GGT ATC TAA TCC-3’), which are specific to the V4 hypervariable region. The ITS region was targeted with the primers ITS1F (5’- CTT GGT CAT TTA GAG GAA GTA A -3’) and ITS2 (5’- GCT GCG TTC TTC ATC GAT GC -3’).

### Bioinformatics analysis

Raw sequences were split according to their unique barcodes and trimmed of the adaptors and primer sequences using QIIME [43]. After removal of low-quality sequences, forward and reverse sequences for each sample were merged. The sequences retained for each sample were processed according to the UPARSE pipeline to generate an operational taxonomic unit (OTU) table [44]. Finally, a representative sequence for each OTU was selected [44] and classified using the RDP classifier [45] against the RDP Bacterial 16S database for bacteria [45] and the UNITE Fungal ITS database for fungi [46]. All raw sequence data have been made available in the NCBI Sequence Read Archive (SRA) database under the accession number SRP239482.

The relative abundance of a given taxonomic group per sample was calculated as the number of sequences affiliated to that group divided by the total number of sequences. Non-metric multidimensional scaling (NMDS) based on a Bray-Curtis dissimilarity matrix was performed and plotted using the R vegan package to explore the differences in microbial communities [47]. Permutational multivariate analysis of variance (PERMANOVA) was conducted to evaluate the effects of fertilizer type on the whole soil microbial community by using the R vegan package [47, 48]. While mantel test was implemented in the R vegan package to identify the correlation between soil microbial community and Fusarium wilt disease incidence [47].

### Quantitative real-time PCR analysis

Quantitative real-time PCR amplifications (qPCR) were used to determine the abundances of total bacteria, fungi, *Fusarium oxysporum*, *Bacillus* and *Pseudomonas* in the bulk soil and banana rhizosphere, according to previously described protocols [49]. Abundances of bacteria and fungi were quantified with primers Eub338F / Eub518R and ITS1f / 5.8s, respectively (Table S1), according to Fierer [50]. Standard curves were generated using 10-fold serial dilutions of a plasmid containing a full-length copy of the 16S rRNA gene from *Escherichia coli* and the 18S rRNA gene from *Saccharomyces cerevisiae*. The abundance of *Fusarium oxysporum* was determined using a SYBR Green assay with the primers FOF1 and FOR1 [51] (Table S1), targeting the rRNA internal transcribed spacer (ITS). A serial dilution from 10^8^ to 10^2^ gene copies μl^-1^ of the ITS gene from the Foc-TR4 strain was used as a standard. The abundance of *Pseudomonas* and *Bacillus* were determined using SYBR Green assays with the primers Ps-for / Ps-rev [52] and Bs16S1 / Bs16SR [53], respectively (Table S1). A serial dilution from 10^8^ to 10^2^ gene copies μl^-1^ of the 16S rRNA gene from *Pseudomonas fluorescens* and *Bacillus subtilis* strains were used as standards. Each assay was performed in triplicate, and the results were expressed as log_10_ values (target copy number g^-1^ soil) prior to further statistical analysis.

### Assay of culturable *Fusarium* and *Bacillus*

To complement the results of the molecular methods described above, we also determined the population densities of culturable *Fusarium* and *Bacillus* in bulk soil and banana rhizosphere samples. This was carried out used using a standard 10-fold dilution plating assay as described by Wang [39]. For enumeration of *Fusarium*, three aliquots (100 μl) per dilution were spread on Komada’s medium [54], and colonies were counted after incubation at 28°C for 5 days. For quantification of *Bacillus* density, three aliquots (100 μl) per dilution were spread on salt V8 agar *Bacillus*-semi-selective medium [55], and plates were incubated at 30°C for 2 days prior to colony counting.

### *Pseudomonas* CFU quantification, strain isolation and identification, and assays of *Fusarium* inhibition, biofilm formation and *Bacillus* attraction

Given the demonstrated role of members of the genus *Pseudomonas* in disease suppression [17, 33], and the results from bacterial community analyses (see below), we tracked the density and functional potential of this genus by cultivation-dependent methods. *Pseudomonas* counts for all samples were determined by 10-fold dilution plating as described by Wang [39]. Three aliquots (100 μl) per dilution were spread on CFC agar *Pseudomonas*-selective medium, and the resulting plates were incubated at 30°C for 3 days prior to colony enumeration. We also isolated *Pseudomonas* strains from the bioorganic fertilizer-treated and organic fertilizer-treated soils after two years of plant growth to compare their potential to inhibit *F. oxysporum* and their ability to produce biofilms. Strains were isolated from the same dilution series described above, using plates with one order of magnitude greater dilution than those used for cell enumeration. A total of 88 *Pseudomonas* isolates (50 and 38 from the OF+W19 and OF treatments, respectively) were purified and identified according to Su [56]. The ability of *Pseudomonas* isolates to inhibit the growth of *F. oxysporum* was tested using a dual culture assay as previously described [57].

We examined biofilm formation of each of the 88 *Pseudomonas* isolates both independently and in co-culture with *B. amyloliquefaciens* W19. Biofilm formation was assayed and quantified as previously described by Ren [58]. Briefly, exponential phase cultures of *Pseudomonas* isolates and *B. amyloliquefaciens* W19 were adjusted to an optical density at 600 nm (OD600) of 0.15 in tryptic soy broth medium and then inoculated into Nunc-TSP plate. The inoculum volumes were 160 ul for TSB and 40 ul of bacterial suspensions (40 μl of W19 or each *Pseudomonas* isolate for monoculture assays and 20 μl of each *Pseudomonas* isolate + 20 μl of W19 for co-culture assays). After 72 h incubation at 30^o^C, biofilm formation was quantified by a modified crystal violet (CV) assay [59, 60]. Interactive effects on biofilm formation were calculated by comparing two-species biofilm results (Abs570 TB) to those of each individual *Pseudomonas* isolate (Abs570 PB), as well as *B. amyloliquefaciens* W19 (Abs570 BB) in monoculture. Results were subsequently presented as follows: Abs570 TB > Abs570 BB and Abs570 TB > Abs570 PB (t-test, *P* < 0.05) = biofilm enhancement [58].

Attraction between the *B. amyloliquefaciens* W19 and *Pseudomonas* isolates was quantified using petri-dish confrontation assays as described by Berendsen [61]. Briefly, each *Pseudomonas* isolate and *B. amyloliquefaciens* W19 was inoculated in 5 mL TSB medium and incubated overnight at 30^o^C at 180 rpm. The optical density of the bacterial cultures was adjusted to 0.1 at 600 nm. Five times 1 μl of these dilutions were inoculated in a diagonal row on both sides of a petri-dish with TSB agar with a multichannel pipet, creating a V-shape of inoculation sites with increasing proximity. Plates were sealed with parafilm and incubated for 7 days at 25^o^C. Colony diameters were measured on an orthogonal to the line dividing the V-shape for calculation of antagonistic effects.

### Effects of selected *Pseudomonas* strains on plant disease levels

We carried out plant-based disease inhibition assays using strain PSE78, which belonged to the most responsive OTU in the OF+W19 and SOF+W19 treatments based upon community sequence analysis (OTU7; see below). This strain also exhibited the strongest *Fusarium* inhibition and strongest stimulation of biofilm formation in co-culture with *B*. *amyloliquefaciens* W19 (see below). We also selected an additional strain, PSE82, which lacked these exceptional qualities to allow comparison. Pot experiments with banana were performed using the following four fertilizer treatments: PSE78, sterile organic fertilizer + strain *Pseudomonas* sp. PSE78; PSE82, sterile organic fertilizer + strain *Pseudomonas* sp. PSE82; SBF, sterile organic fertilizer; and CK, chemical fertilizer to the same nutrient levels as achieved by organic fertilizer amendment. The density of each *Pseudomonas* strain was confirmed to be at least 1.0×10^9^ CFU g^-1^ dry weight of fertilizer at the start of the experiment. Experimental design and conditions were identical to those used in the main mesocosm experiment described above.

### Effects of *Bacillus*-*Pseudomonas* co-culture on FOC density

Banana tissue culture seedlings were cultivated in Erlenmeyer flasks and watered with modified strength sterile Hoagland solution. After two weeks, seedlings were transferred to 400-mL pots filled with a sterile substrate pre-inoculated with *B. amyloliquefaciens* W19, *Pseudomonas* sp. PSE78 or *Pseudomonas* sp. PSE82, or a combination of W19 mixed with either PSE78 or PSE82. In all cases, the final inoculation density was 1×10^8^ CFU/g of substrate. The pots (10 replicates per treatment with 3 times experimental repeated) were placed on small saucers, watered with modified strength Hoagland solution, randomly placed in trays and transferred to a growth chamber (28^o^C average temperature, 80% relative humidity, 16 h light/8 h dark). After thirty days, all plants were transplanted into a new sterile substrate. Banana plants were then inoculated with a *Fusarium oxysporum* f. sp. *cubense* (FOC) spore suspension (final density of 1×10^4^ spores/g of substrate as describe above) or a mock suspension. Disease severity was quantified by counting the density of FOC colonizing banana plant roots three weeks after FOC inoculation. FOC, *Bacillus* and *Pseudomonas* densities in the banana roots were determined by suspending approximately 0.1 g of root of eight replicate pots per treatment and plating a dilution series on Komada’s medium, V8 agar *Bacillus*-semi-selective medium, and CFC agar *Pseudomonas*-selective medium as described above, respectively. Fig. S11 provides a schematic representation of this experiment.

### Statistical analyses

All statistical analyses were performed by using the IBM SPSS 20.0 software program (IBM Corporation, New York, USA) and R software programs (Version 3.5.0). All statistical tests performed in this study were considered significant at *P* < 0.05. To determine significant differences, unpaired t-tests and one-way ANOVA were performed. Testing of linear discriminant analysis effect size (LEfSe) was performed to identify significant differences in bacterial and fungal taxa between fertilization regimes [62]. The Kruskal-Wallis (KW) sum-rank test was used in LEfSe analysis to detect the features with significantly different abundances between assigned classes, and linear discriminant analysis (LDA) was then performed to estimate the effect size of each differentially abundant taxon [62]. Spearman's rank correlation coefficients between the relative abundance of OTUs and Fusarium wilt disease incidence were calculated in R software. *P*-value adjustments for multiple comparisons were performed using the false discovery rate (FDR) correction [63]. Fold change of each OTU in treatments with the biocontrol agent (OF+W19 and SOF+W19) relative those without the biocontrol agent (OF and SOF) was calculated using the following formula: (B-N)/N, B is the relative abundance of a given OTU in *Bacillus* positive (OF+W19 and SOF+W19) samples and N represents the relative abundance of that OTU in *Bacillus* negative (OF and SOF) samples [64]. Structural equation modelling (SEM) was applied to evaluate the direct and indirect contributions of soil microbial community (bulk and rhizosphere soil) and *F. oxysporum* pathogen density to disease incidence [65]. The SEM fitness was examined on the basis of a non-significant chi-square test (*P* > 0.05), the goodness-of-fit index (GFI), and the root mean square error of approximation (RMSEA) [66, 67]. Model was fit using the lavaan package in R software [68]. The linear regression analyses relating disease incidence to the selected microbial taxa were conducted using the basicTrendline package in R software.

## Results

### Disease incidence

**Fig. 1.**
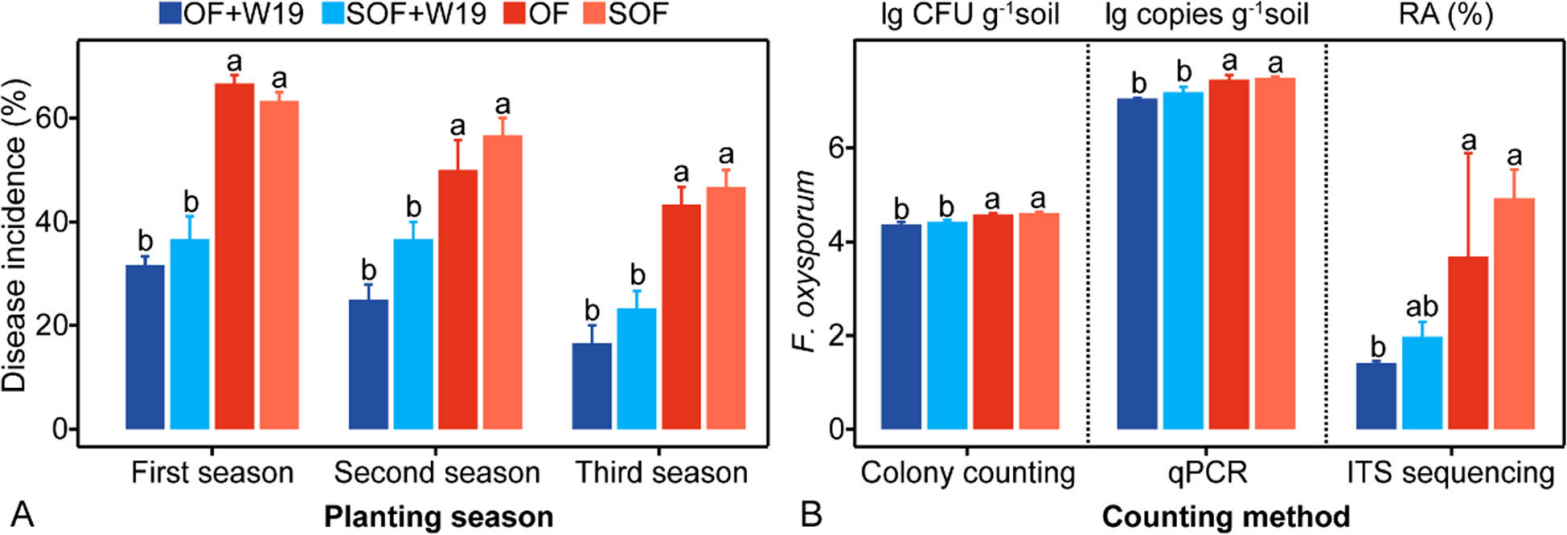
(A) Disease incidence of banana Fusarium wilt in the four fertilizer treatments. (B) Abundance of cultivable, total and relative abundance of *F. oxysporum* in the second season rhizosphere soil. OF+W19 = Bio-organic fertilizer containing *B. amyloliquefaciens* W19, SOF + W19 = sterilized bio-organic fertilizer inoculated with *B*. *amyloliquefaciens* W19, OF = Organic fertilizer, SOF = Sterilized organic fertilizer. Different letters above the bars indicate significant differences at the 0.05 probability level according to the Duncan test (n=3).

In all three seasons of the mesocosm experiment, both bio-fertilizer treatments (OF+W19 and SOF+W19) (Duncan test, *P* < 0.05) reduced banana Fusarium wilt disease incidence, with OF+W19 showing the lowest disease incidence in each season, as compared to the organic fertilizer treatments (SOF and OF) (Fig. 1a). There was no significant difference of disease incidence between the SOF and OF treatments (Duncan test, *P* > 0.05) and between the OF+W19 and SOF+W19 treatments (Duncan test, *P* > 0.05).

From the amplicon sequencing data recovered from soils after the second growing season, we also detected a lower relative abundance of *F. oxysporum* (Duncan test, *P* < 0.05) in the rhizospheres of the OF+W19 and SOF+W19 treatments as compared to those of the SOF and OF treatments (Fig. 1b). This finding was supported by quantitative colony counting and qPCR analyses (Fig. 1b). Disease incidence positively correlated with *F. oxysporum* abundance in rhizosphere soil as determined by all three methods (*P* < 0.001, *P* < 0.001, *P* = 0.002, Fig. S1B), and a positive correlation was found between disease incidence and cultivable *F. oxysporum* abundance in bulk soil (*P* = 0.04, Fig. S1A).

### Microbial community composition

**Fig. 2.**
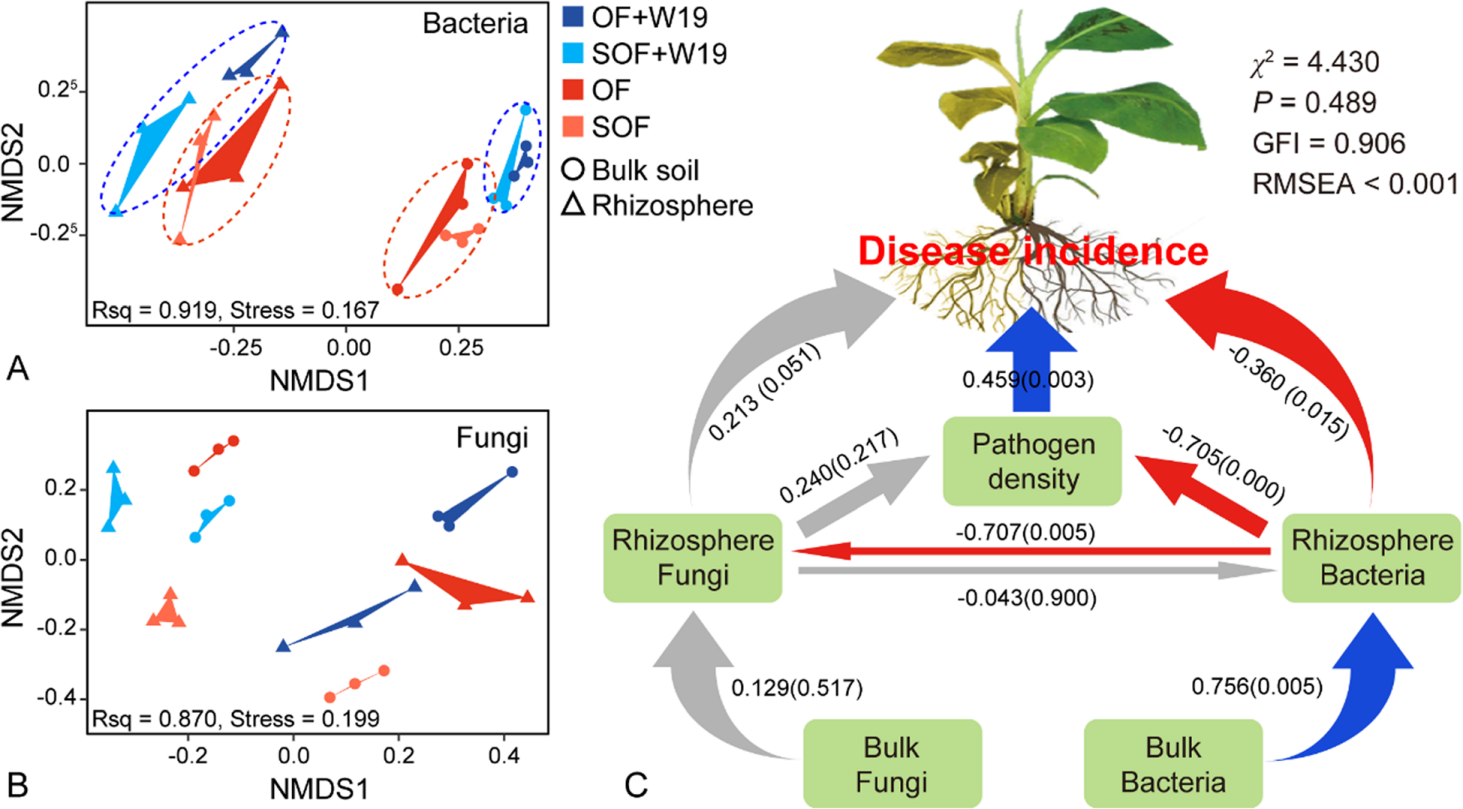
Non-metric multidimensional scaling (NMDS) ordinations of bacterial (A) and fungal (B) community composition across all bulk and rhizosphere soil samples. OF+W19 = Bio-organic fertilizer containing *B. amyloliquefaciens* W19, SOF+W19 = sterilized bio-organic fertilizer inoculated with *B*. *amyloliquefaciens* W19, OF = Organic fertilizer, SOF = Sterilized organic fertilizer. (C) Structural equation model of incorporating bacterial and fungal community structure, *Fusarium* pathogen density and banana *Fusarium* wilt disease incidence. The path analysis numbers adjacent to arrows indicate the relationship’s effect size and the associated bootstrap *P*-value. Blue and red arrows indicate positive and negative relationships, respectively. Paths with non-significant coefficients are presented as gray lines.

Detailed Illumina Miseq sequencing results of microbial community *alpha* diversity are shown in supplementary materials (Fig. S11, S12 and S13). Non-metric multidimensional scaling (NMDS) revealed significant differences in bacterial (*P* (bulk) = 0.007, *P* (rhizosphere) = 0.038), but not the fungal community composition (*P* (bulk) = 0.106, *P* (rhizosphere) = 0.217) across the different treatments for both bulk and rhizosphere soils (Fig. 2, Table S2). Overall, bacterial community composition from the OF+W19 and SOF+W19 treatments were rather similar and clearly distinct from the OF and SOF treatments along the first axis (NMDS1) in bulk soil (*P* = 0.012) (Fig. 2a). The OF and SOF treatments grouped together and differed from the OF+W19 and SOF +W19 treatments along the first axis (NMDS1) in rhizosphere soil (*P* = 0.027) (Fig. 2a). For fungi, OF and SOF+W19 treatments grouped together and were distinct from the OF+W19 and SOF treatments in bulk soil (*P* < 0.001) (Fig. 2b). The OF+W19 and OF treatments were similar to each other and distinct from the SOF+W19 and SOF treatments along the first axis (NMDS1) in the rhizosphere (*P* = 0.005) (Fig. 2b). Bacterial community composition could be grouped into the two bio-fertilizer (OF+W19 and SOF+W19) treatments and the two organic fertilizer (OF and SOF) treatments for both bulk and rhizosphere soils (*P* (bulk) = 0.007, *P* (rhizosphere) = 0.034), while fungal communities did not show clear patterns with respect to the different fertilization regimes (*P* (bulk) = 0.093, *P* (rhizosphere) = 0.319).

Structural equation modeling (path analysis) (Fig. 2c) showed that the strongest driver explaining disease was pathogen density (r = 0.459, *p* = 0.003), which was negatively affected by rhizosphere bacterial community composition (r = -0.360, *p* = 0.015). Bulk soil bacterial community structure also determined rhizosphere bacterial community composition to a large extent (r = 0.756, *p* = 0.005). In addition, we evaluated the correlation between bulk and rhizosphere soil microbiota with Fusarium wilt disease incidence and found that only rhizosphere bacterial community composition correlated with disease incidence (Mantel test, *P* = 0.01; PERMANOVA, *P* = 0.008, Table S3).

### Responsive microbial taxa

**Fig. 3.**
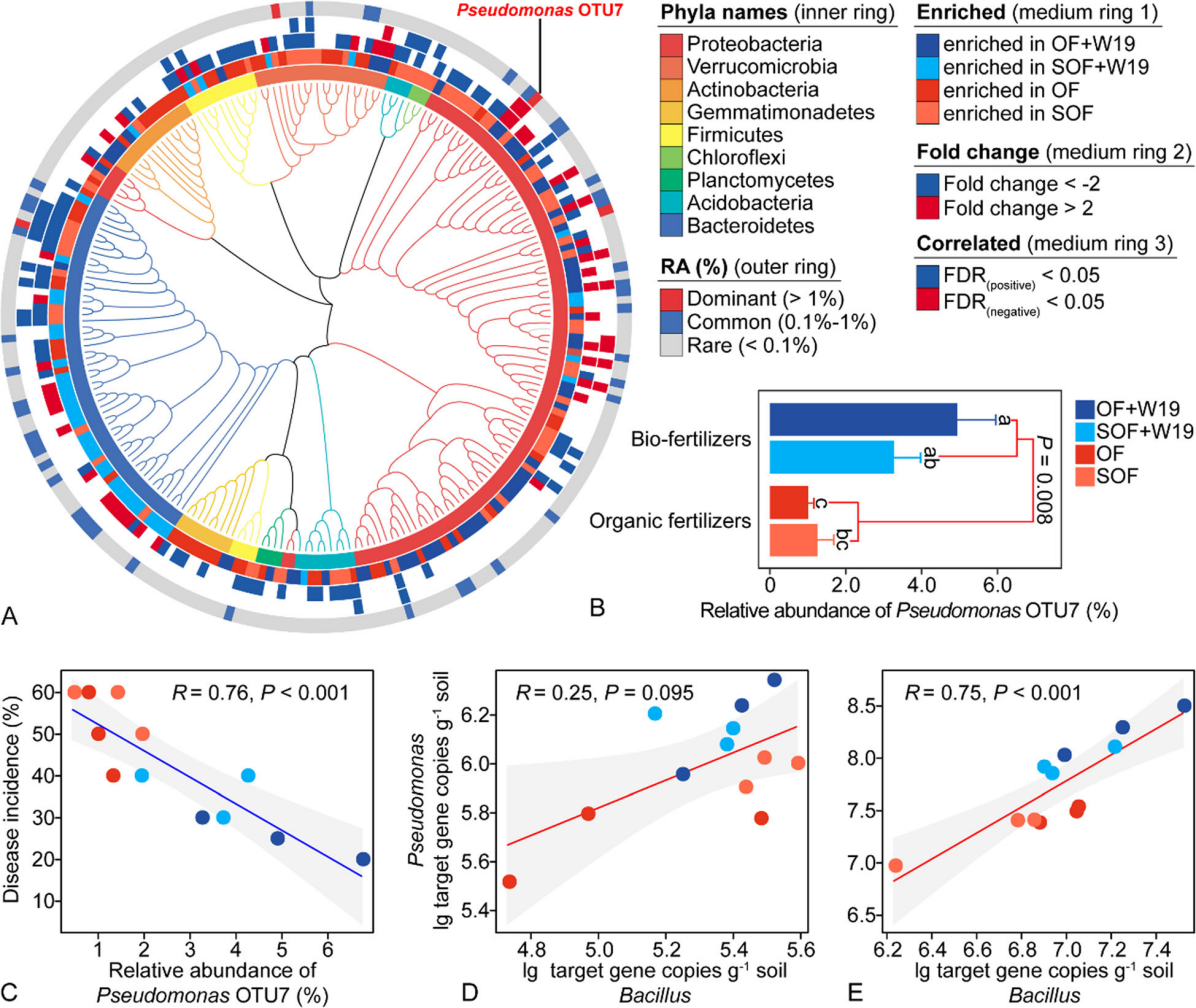
(A) Cladogram showing phylogenetic relationships between 233 rhizosphere soil bacterial OTUs. Leaf labels indicate representative sequence IDs. Rings, from the inner to the outside circles, represent: 1) phylum-level taxonomy of OTUs; 2) OTUs responding significantly to the four treatments (LDA > 2); 3) fold change of OTUs; 4) correlations between OTU relative abundance and disease incidence; and 5) variable pattern of OTU relative abundance. (B) Relative abundance of *Pseudomonas* OTU7 across the different treatments. (C) Linear regression relationship between the relative abundance of *Pseudomonas* OTU7 and disease incidence. Linear regression relationship between population densities of total *Bacillus* and *Pseudomonas* in bulk (D) and rhizosphere soil (E). OF+W19 = Bio-organic fertilizer containing *B. amyloliquefaciens* W19, SOF+W19 = sterilized bio-organic fertilizer inoculated with *B*. *amyloliquefaciens* W19, OF = Organic fertilizer, SOF = Sterilized organic fertilizer. Different letters above the bars indicate significant differences at the *P* < 0.05 probability level, according to the Duncan test (n=3)

We further examined which bacterial OTUs were correlated with specific fertilization treatments and the level of banana wilt disease. Based on linear discriminant analysis (LDA), 233 rhizosphere bacterial OTUs differed between fertilization regimes. Of those, 43 OTUs were enriched in bio-organic fertilizer treatments (> 2-fold increase in abundance) compared with organic fertilizer treatments. Spearman's rank correlation analysis found 34 OTUs linked with disease incidence (FDR < 0.05) (Fig. 3a). Among those responsive microbial taxa, the dominant taxon OTU7, assigned as a *Pseudomonas* sp., showed a particularly striking pattern, prompting a more detailed examination of this OTU. *Pseudomonas* OTU7 had the highest relative abundance in the biofertilizer treatments, averaging 4.94% and 3.27% of the total bacterial community for OF+W19 and SOF+W19, respectively (Fig. 3b). In contrast, this OTU only represented 1.00% and 1.25% of the total bacterial community in the OF and SOF treatments, respectively (Fig. 3b). In addition, *Pseudomonas* OTU7 was negatively correlated with Fusarium wilt disease incidence (*P* < 0.001) (Fig. 3c).

### Relationship between the inoculated biocontrol *Bacillus* and indigenous *Pseudomonas* density

To investigate if the genus *Pseudomonas* had an overall response to the input of the biocontrol strain via bioorganic fertilizer, we examined the abundance of *Pseudomonas* and *Bacillus* in bulk and rhizosphere soils. Higher total and cultivable *Bacillus* and *Pseudomonas* abundances were detected in the two bio-fertilizer treatments (OF+W19 and SOF+W19) as compared to the two organic fertilizer treatments in the rhizosphere (Duncan test, *P* < 0.05; Fig. S2A, B). The OF+W19 and SOF+W19 treatments also increased the abundance of cultivable *Bacillus* and total *Pseudomonas* in the bulk soil (Duncan test, *P* < 0.05; Fig. S2A, B). We observed a positive relationship between total and cultivable *Bacillus* and *Pseudomonas* for rhizosphere soils (*P* < 0.001, *P* < 0.001, Fig. 3e, S3B) and a not significant trend for bulk soils (*P* = 0.095, *P* = 0.297; Fig. 3d, S3A). Furthermore, disease incidence was negatively correlated with total and cultivable *Pseudomonas* (*P* = 0.002, *P* = 0.007) and *Bacillus* (*P* = 0.002, *P* < 0.001) population densities in the rhizosphere (Fig. S4B, D), while cultivable *Bacillus* (*P* = 0.03) and total *Pseudomonas* (*P* = 0.04) showed the same trends in bulk soil (Fig. S4A, C).

### *Pseudomonas* isolates and their properties of *Fusarium* inhibition, biofilm formation and *Bacillus* attraction

**Fig. 4.**
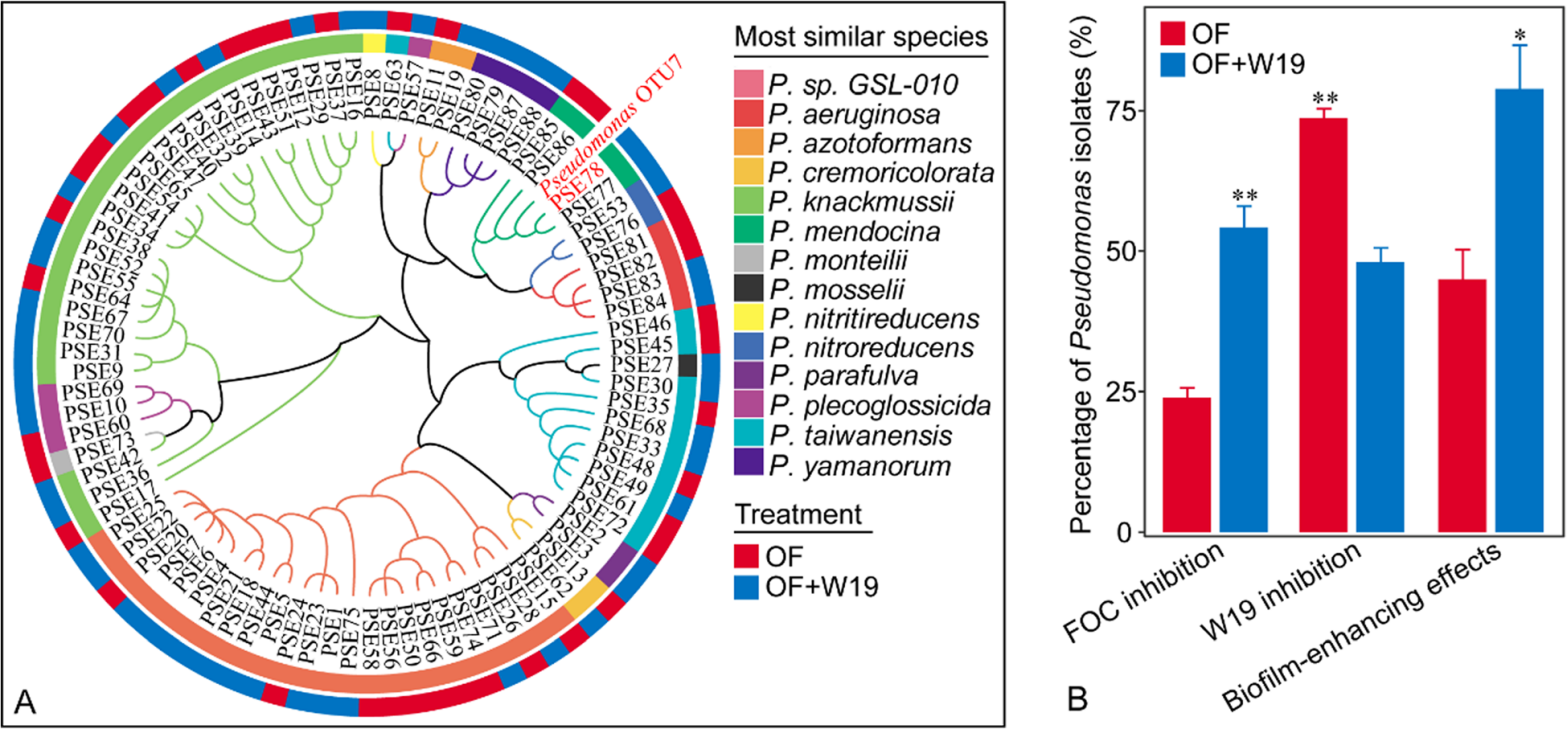
(A) Cladogram showing phylogenetic relationships between 88 rhizosphere soil *Pseudomonas* spp. as well as OTU7. Leaf labels indicate representative sequence IDs. The inner rings indicates the species-level taxonomy, and the outer ring represents the soil from which the strain was isolated. (B) The percentage of *Pseudomonas* isolates with FOC inhibition ability or with *B. amyloliquefaciens* W19 inhibition ability in our dual challenge assays, or with biofilm-enhancing effects in co-culture biofilm assays with W19 (t-test, mean SD, n = 3; *P < 0.05, **P < 0.01). OF+W19 = Bio-organic fertilizer containing *B. amyloliquefaciens* W19, OF = Organic fertilizer

A total of 88 *Pseudomonas* strains were randomly isolated and identified from rhizosphere soil microbiomes amended with bio-organic and organic fertilizers, representing 14 distinct phylogenic groups based on 16S rRNA gene sequences (Fig. 4a). A total of 36 of these isolates showed antagonistic activity against the Fusarium wilt pathogen *Fusarium oxysporum* f. sp. *cubense* (FOC), with a greater proportion of *Pseudomonas* isolates from the bio-organic fertilizer treatment (OF+W19) showing such inhibition compared to isolates recovered from the organic fertilizer treatment (t-test, *P* < 0.01, Fig. 4b). 52% of *Pseudomonas* isolates from the rhizosphere soil of OF+W19 showed no influence on *B*. *amyloliquefaciens* W19, while 74% of *Pseudomonas* isolates from the rhizosphere soil amended with organic fertilizer (OF) showed an inhibition of W19 (t-test, *P* < 0.01, Fig. 4b). In monocultures system, there was no significant difference between biofilm formation of *Pseudomonas* isolates from the OF+W19 and OF treatments (Fig. S5A). In co-culture systems combining *Pseudomonas* isolates together with W19, average biofilm formation was greater than that observed for the *Pseudomonas* isolates in monoculture (Fig. S5B, Wilcoxon-test, *p* < 0.001). In total, *Pseudomonas* isolates from the OF+W19 treatment were much more effective at improving dual-species biofilm formation (Fig. S5A, Wilcoxon-test, *p* < 0.001). A higher proportion of *Pseudomonas* isolates from this treatment displayed a stimulatory effect on biofilm formation with W19 (t-test, *P* < 0.05, Fig. 4b), while fewer strains showed antagonism against W19 (Fig. S5C, Wilcoxon-test, *p* < 0.001).

### Disease suppression ability of *Pseudomonas* strain PSE78 and its potential interactions with *B*. *amyloliquefaciens* W19

**Fig. 5:**
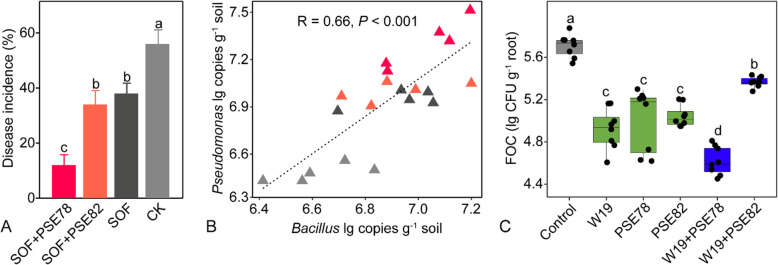
(A) Disease incidence of banana Fusarium wilt in soils treated with sterilized organic fertilizer inoculated with either *Pseudomonas* sp. PSE78 or PSE82 as compared to sterilized organic fertilizer (SOF) and chemical fertilizer (CK) treatments. (B) Linear regression between the population densities of *Bacillus* and *Pseudomonas* in the banana rhizosphere soil. Symbol colors correspond to the treatment designations given in panel A. (C) Boxplot showing the number of FOC colonized on plant roots growing on the indicated pre-conditioned substrates. Different letters indicate a significant difference at the 0.05 probability level according to the Duncan test (n=8)

We examined the ability of *Pseudomonas* strain PSE78 to suppress FOC disease in banana using a pot-based experiment. This isolate was chosen because it showed the strongest antagonistic activity against *F. oxysporum* (Fig. S6B and C), had positive interactions with *B. amyloliquefaciens* W19 in biofilm formation (Fig. S6A), and displayed 99% sequence identity with the most responsive OTU (see above), OTU7 (Fig. 4a). For sake of comparison, we also examined a strain without these exceptional qualities, *Pseudomonas* isolate PSE82. *Pseudomonas* strain PSE78 showed a strong ability to suppress Fusarium wilt disease (Fig. 5a). Compared with CK and other treatments, the PSE78 treatment showed lower disease incidence (Duncan test, *P* < 0.05) with an average value of 12%, while no difference (Duncan test, *P* > 0.05) in disease incidence was found when comparing the effects of *Pseudomonas* strain PSE82 with the SOF treatment (Fig. 5a). In addition, higher *Pseudomonas* and lower *F. oxysporum* population densities were detected in the rhizosphere soil of the PSE78 treatment (Duncan test, *P* < 0.05; Fig. S7 and Fig. S8A). There were also positive and negative relationships between disease incidence and the respective population densities of *F. oxysporum* and *Pseudomonas* in the rhizosphere (*P* < 0.001, *P* < 0.001; Fig. S9E and F).

To investigate whether there was a general correlation between *Pseudomonas* and *Bacillus*, we further examined the abundance of indigenous *Bacillus* in the rhizosphere. Higher *Bacillus* population densities in the rhizosphere were detected in the PSE78 treatment as compared to other treatments (Duncan test, *P* < 0.05, Fig. S8B). *Bacillus* and *Pseudomonas* populations were positively correlated (*P* < 0.001, Fig. 5B and Fig. S9H), and both were negatively correlated with disease incidence (*P* < 0.001, *P* < 0.001, Fig. S9F and G).

We further examined if interactions between *Pseudomonas* sp. PSE78 and *B. amyloliquefaciens* W19 synergistically enhanced suppression of *Fusarium oxysporum* f. sp. *cubense* (FOC) by using a modified dual challenge assay (Fig. S6B). Indeed, isolate PSE78 showed a synergistic effect on FOC inhibition in combination with W19, as combination of the two strains yielded a greater bacteriostatic area than predicted by their individual behaviors (Fig. S6B and C). We also extended this examination to investigate possible synergistic effects between PSE78 and W19 on the suppression of FOC in soil, again using PSE82 for comparison. Although all three strains reduced FOC density to some extent when applied individually, the largest reduction in FOC density was observed when PSE 78 and W19 were co-inoculated in the experimental soil (Fig. 5c). Interestingly, the combination PSE82 with W19 was less effective in inhibiting the pathogen than either of these strains individually (Fig. 5c).

## Discussion

In this study, we examined the impacts of bioorganic fertilizer on disease suppression within a continuous banana monoculture cropping system in a naturally diseased soil. We imposed treatments with sterilized or non-sterilized organic fertilizer both either containing or lacking inoculation with *B*. *amyloliquefaciens* W19, a well-studied biocontrol agent of Fusarium wilt disease [36, 39]. Our objective was to disentangle the relative contribution of the organic substrate addition, fertilizer microbiome and inoculated biocontrol strain on disease suppression. We found that re-inoculation of sterilized compost with *B*. *amyloliquefaciens* W19 yielded a comparable degree of disease suppression as observed for the intact bioorganic fertilizer treatment. We further found that the total effect of bioorganic fertilizer was the sum of direct pathogen inhibition by the biocontrol strain as well as indirect effects due to reshaping of the resident soil microbial community, with a particularly important role of specific *Pseudomonas* populations.

### Relative importance of different components of bio-organic fertilizer

Our results indicate that the biocontrol agent *B*. *amyloliquefaciens* W19 used to produce the bioorganic fertilizer was critical to effective suppression of Fusarium wilt disease. In contrast, the pure physical / chemical properties of the fertilizer and the microbiome resident to the fertilizer substrate did not have any detectable effects on the level of disease suppression. These findings are in line with previous findings that show that organic matter amendments alone are often ineffective or even conducive to plant pathogen infection [69], yet addition of a suitable biological control agent (such as *Bacillus* spp., *Trichoderma* spp., etc.) to organic fertilizer can effectively reduce *Fusarium* pathogens and thereby control plant disease [28, 36]. Although *B*. *amyloliquefaciens* W19 and indigenous *Bacillus* could not be separately quantified in our experiments, the higher population densities of *Bacillus* in bio-organic fertilizers treatments (sterilized or non-sterilized organic fertilizer inoculated with *B*. *amyloliquefaciens* W19) suggests that the inputs of the biological control agent are involved in enhanced disease suppression ability via direct antagonistic effects on the plant pathogen [39, 70].

### Impact of biocontrol agent on the resident soil community as pathway to disease suppression

We found that all four fertilizer inputs induced specific changes in the microbiome structure, a result that is similar to previous findings tracking responses to different fertilization management regimes [71, 72]. As also found previously, bacterial communities appeared to be more affected by fertilizer treatments than fungal communities [73]. The changes we observed in response to fertilizer could also be linked with the level of disease suppression (Fig. 2c), implying that bacterial community changes had important functional consequences [38, 61, 74, 75].

We observed that the fertilizer treatments containing the biocontrol agent resulted in a striking increase in the relative density of the genus *Pseudomonas* spp., with one specific dominant OTU in particular, OTU7, showing the strongest response. A number of well-known plant growth-promoting bacteria belong to this genus, and *Pseudomonas* species have previously been linked to disease suppression [17, 32, 33, 35]. Interestingly, we found the population densities of *Bacillus* and *Pseudomonas* to be correlated with each other, and negatively correlated with *F*. oxysporum density and wilt disease. We explored this correlation further, finding a lower proportion of *Pseudomonas* strains showing antagonistic activity against *B*. *amyloliquefaciens* W19 in the bio-organic fertilizer treatment as compared to the control (Fig. 4b). Notably, we found that several *Pseudomonas* spp., whose growth was not inhibited by *B*. *amyloliquefaciens* W19, showed high antagonism toward the pathogen via antifungal metabolites [15, 32]. It is thus possible that the effects of *Bacillus* inoculation were further reinforced by the enrichment of antagonistic *Pseudomonas* present in the resident microbiome leading to effective disease suppression.

### Potential mechanisms behind combined effect of *Bacillus* and *Pseudomonas* on pathogen suppression

*Pseudomonas* spp. are widely used biocontrol agents used to combat soil-borne plant diseases [33, 35], and they have often identified as important members of microbiomes from naturally occurring disease suppressive soils [15, 17, 33]. The diversity of *Pseudomonas* spp. is, however, high in natural soils [76] and not all *Pseudomonas* spp. have such impacts on disease suppression, as we have also found in our study. We zoomed in specifically on PSE 78, as a representative of *Pseudomonas* OTU7. While our studies cannot demonstrate that the interactions between *Pseudomonas* OTU7 and *B*. *amyloliquefaciens* W19 are responsible for the level of disease suppression found in our main experiment, our data (Fig. 5c) and other reports have shown that disease suppression can often be attributed to the combined action of bacterial populations that far less effect on disease suppression individually [25, 77, 78]. Our findings also show the importance of plant-beneficial *Bacillus* in disease suppression through positive interactions with this specific *Pseudomonas* leading to increased biofilm formation and root colonization. In particular, we found that isolate *Pseudomonas* PSE 78, but not PSE 82, can interact synergistically in biofilm formation in co-culture with *B*. *amyloliquefaciens* W19, suggesting an important role in community assembly at the root-microbiome interface (Fig. 4b). A similar mode of induced plant pathogen resistance by interactive biofilm formation was recently reported [61]. Other studies have also suggested that bacterial interspecific interactions can enhance biofilm formation and microbial fitness [58, 79], thereby potentially triggering microbial root colonization and subsequent plant disease resistance [61, 80–82]. Our data supports the hypothesis that the combined action of specifically stimulated *Pseudomonas* species (PSE 78 not PSE 82) together with *B*. *amyloliquefaciens* W19 leads to the marked decrease in the density of FOC within the root zone of banana (Fig. 5c).

Our findings suggests that the assembly of multispecies biofilms composed of *Bacillus* spp. and *Pseudomonas* spp. at the root-microbiome interface can help shield the plant from pathogen infection [80, 83, 84]. Other mechanisms involved in the observed pathogen control might involve quorum sensing (QS) signals, siderophore-mediated interactions and systemically induced root exudation of metabolites (SIREM) [85–87]. Here, we report that the inoculation of specific biological control agents can help stimulate specific beneficial plant-associated microbes that together have the potential to protect the plants against pathogen attack. Future investigations, potentially using transcriptomic and proteomic approaches, would be necessary to delineate the exact nature of the molecular dialog between these rhizosphere partners and how their combined action serves to confer disease suppression.

## Conclusions

**Fig. 6.**
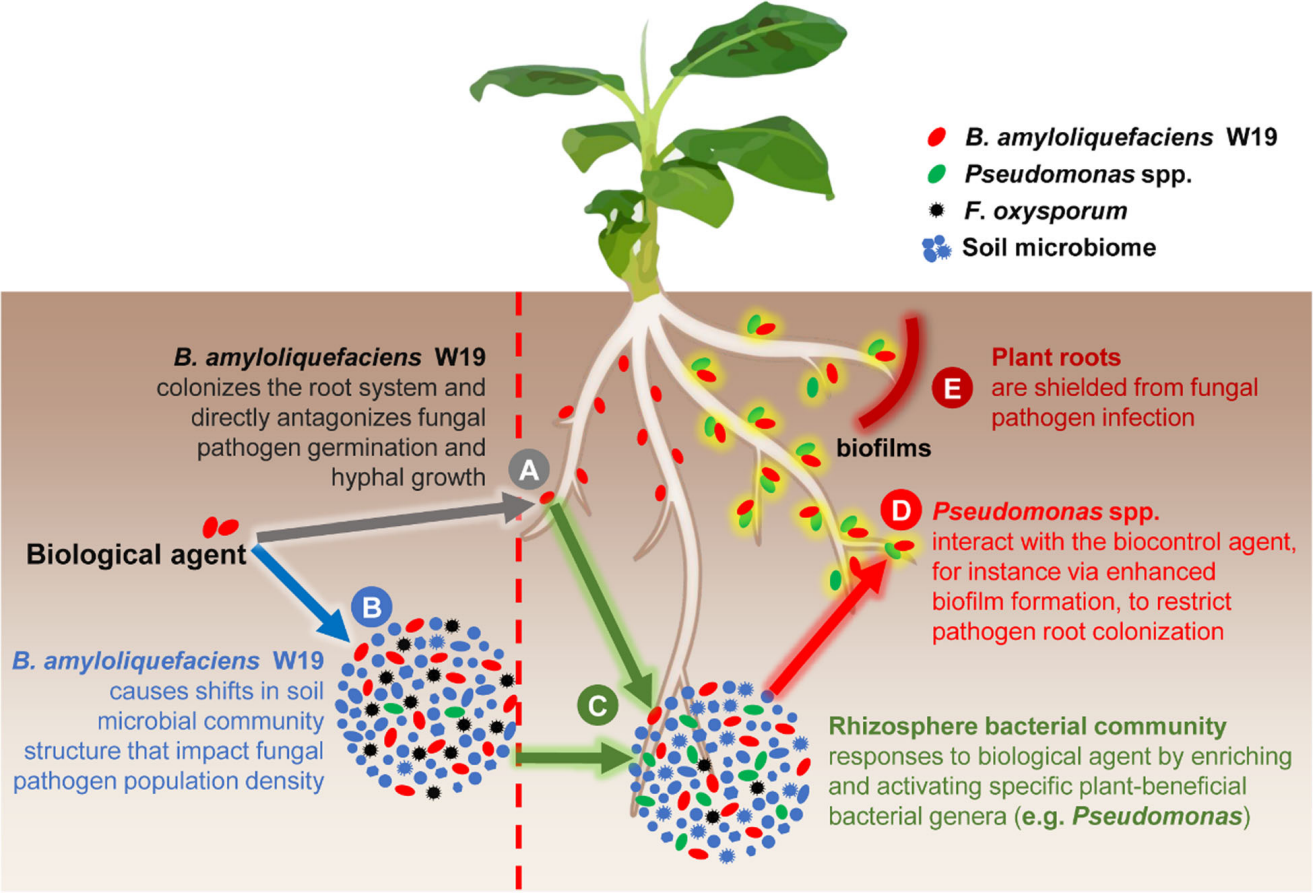
Conceptual model illustrating the proposed sequence of events (A thru E) taking place in the rhizosphere of plants grown in bio-organic fertilizer-amended soil. Depicted are the biofertilizer-induced changes in microbial community composition and activities that restrict fungal pathogen growth and subsequent plant infection

We have summarized the results of our experiments in a conceptual model (Fig. 6) depicting the mode of action by which bio-organic fertilizer application leads to the suppression of Fusarium wilt disease. The biological agent, *B*. *amyloliquefaciens* W19, has the ability to establish in the rhizosphere where it can (A) directly inhibit pathogen growth; (B) induce changes in especially the bacterial part of the microbiome with negative consequences on pathogen density; (C) co-activate specific beneficial plant-associated microorganisms present in the rhizosphere microbiome, (D) potentially leading to the activation of multispecies biofilm formation with a specific plant-beneficial bacterial genus (such as *Pseudomonas* spp.), and thereby, (E) directly or indirectly, restrict the ability of the fungal pathogen to infect the plant root. Our experimental design allowed us to disentangle the importance of different components of bio-organic fertilizer in yielding effective disease suppression. We further could gain insight into how indigenous *Pseudomonas* spp. are promoted in soils leading to a combined action together with the biocontrol agent to achieve disease suppression. These insights provide new mechanistic underpinnings to how specific management measures lead to disease suppression, opening up new opportunities for more effective applications. For instance, future biocontrol strategies might involve co-inoculating synergistically interacting bacterial species to specifically promote soil function. Also, potential biocontrol strains might also be screened not only for their ability to antagonize the pathogen of interest, but also for their ability to stimulate potentially synergistic resident populations.

## Supplementary information

**Additional file 1: Table S1** Sequences of oligonucleotide primers required for quantitative PCR. **Table S2** PERMANOVA results for bacterial and fungal community structure at the OTU level. **Table S3** Spearman's correlations between Fusarium wilt disease incidence and microbiota determined by Mantel test. **Fig. S1**. Linear regression between the abundances of cultivable and total *F*. *oxysporum* and relative abundance of *Fusarium* with disease incidence for bulk (A) and rhizosphere (B) soil. **Fig. S2**. The abundance of total and cultivable *Bacillus* (A) and *Pseudomonas* (B) in banana bulk and rhizosphere soil. **Fig. S3**. Linear regression between the abundances of cultivable *Bacillus* and *Pseudomonas* in bulk soil (A) and rhizosphere soil (B). **Fig. S4**. Linear regression between the abundances of total and cultivable *Bacillus* (A, B) and *Pseudomonas* (C, D) with disease incidence for bulk and rhizosphere soil, respectively. **Fig. S5** (A) Biofilm formation of *Pseudomonas* isolates and *B. amyloliquefaciens* W19 in monocultures and *Pseudomonas-Bacillus* cocultures systems for isolates from OF+W19 and OF treatments; (B) Biofilm formation of *Pseudomonas* isolates and *B. amyloliquefaciens* W19 in monocultures and *Pseudomonas-Bacillus* cocultures systems; and (C) Biofilm formation of *Pseudomonas* isolates and *B. amyloliquefaciens* W19 in *Pseudomonas-Bacillus* cocultures systems for isolates from antagonistic group (antagonistic relationship between *Pseudomonas* spp. and W19) or non-antagonistic group (non-antagonistic relationship between *Pseudomonas* spp. and W19). **Fig. S6**. Biofilm formation of *Pseudomonas* strain PSE78 or PSE82 with *B. amyloliquefaciens* W19 in monocultures and *Pseudomonas-Bacillus* co-culture systems (A). *Pseudomonas*-*Bacillus*-*Fusarium oxysporum* f. sp. *cubense* (FOC) interaction model (B). The interactions between *Fusarium oxysporum* f. sp. *cubense* (FOC), *B*. *amyloliquefaciens* W19 and *Pseudomonas* sp. PSE78 or PSE82 (C). **Fig. S7**. The abundance of total and cultivable *F. oxysporum* in banana bulk and rhizosphere soil treated with sterilized organic fertilizer inoculated with either *Pseudomonas* PSE78 or PSE82 as compared to sterilized organic fertilizer (SOF) and chemical fertilizer (CK) treatments. **Fig. S8**. The abundance of total and cultivable *Pseudomonas* and *Bacillus* in banana bulk and rhizosphere soil treated with sterilized organic fertilizer inoculated with either *Pseudomonas* PSE78 or PSE82 as compared to sterilized organic fertilizer (SOF) and chemical fertilizer (CK) treatments. **Fig. S9**. Linear regression between total and cultivable *Fusarium*, *Pseudomonas* and *Bacillus* with disease incidence in bulk soil (A, B, and C) and rhizosphere soil (E, F, and G). Linear regression between total and cultivable *Bacillus* and *Pseudomonas* in bulk soil (D) and rhizosphere soil (H). **Fig. S10**. Schematic representation of the experimental design for testing combined impacts of PSE strains and *Bacillus amyloliquefaciens* W19 on plant disease. (I) Sterile banana seedlings were inoculated with *Bacillus amyloliquefaciens* W19, *Pseudomonas* sp. PSE78 (PSE78), or *Pseudomonas* sp. PSE82 (PSE82), or a combination of W19 and PSE78 (W19+PSE78) or W19 and PSE82 (W19+PSE82) or mock treated (Control). (II) Thirty days after inoculation, all plants were transplanted into a new sterile substrate, (III) after which the banana plants were either inoculated with *Fusarium oxysporum* f. sp. *cubense* (FOC) spore suspension or given a mock inoculation. Disease severity was quantified by counting the number of FOC that colonized on plant roots after 3 weeks of inoculation. Supplementary results**. Fig. S11**. Rarefaction curves of 16S rRNA genes (bacteria) and ITS sequences (fungi) at 97 % similarity levels of the bulk and rhizosphere soil. OF+W19 = Bio-organic fertilizer containing *B*. *amyloliquefaciens* W19, SOF+W19 = sterilized bio-organic fertilizer inoculated with *B*. *amyloliquefaciens* W19, OF = Organic fertilizer, SOF = Sterilized organic fertilizer. Fig. S12. The relative abundance of bacterial (dominate bacterial phyla) and fungal phyla in bulk and banana rhizosphere soils. OF+W19 = Bio-organic fertilizer containing *B*. *amyloliquefaciens* W19, SOF+W19 = sterilized bio-organic fertilizer inoculated with *B*. *amyloliquefaciens* W19, OF = Organic fertilizer, SOF = Sterilized organic fertilizer. **Fig. S13.** Bacterial and fungal richness (Sobs) (A) and diversity (Shannon) (B) in bulk and banana rhizosphere soils. Different letters indicate significant difference at the 0.05 probability level according to the Duncan test. OF+W19 = Bio-organic fertilizer containing *B*. *amyloliquefaciens* W19, SOF+W19 = sterilized bio-organic fertilizer inoculated with *B*. *amyloliquefaciens* W19, OF = Organic fertilizer, SOF = Sterilized organic fertilizer.

## Data Availability

All raw sequence data have been made available in the NCBI Sequence Read Archive (SRA) database under the accession number SRP239482.
