# Supplementary material for: Bio-organic fertilizers stimulate indigenous soil Pseudomonas populations to enhance plant disease suppression
Source: Microbiome. 2020 Sep 22;8:137. doi: 10.1186/s40168-020-00892-z (PMC7510105; doi:10.1186/s40168-020-00892-z)
Supplement: Supplementary file 2 — Additional file 1: Table S1 Sequences of oligonucleotide primers required for quantitative PCR. Table S2 PERMANOVA results for bacterial and fungal community structure at the OTU level. Table S3 Spearman's correlations between Fusarium wilt disease incidence and microbiota determined by Mantel test. Fig. S1. Linear regression between the abundances of cultivable and total F. oxysporum and relative abundance of Fusarium with disease incidence for bulk (A) and rhizosphere (B) soil. Fig. S2. The abundance of total and cultivable Bacillus (A) and Pseudomonas (B) in banana bulk and rhizosphere soil. Fig. S3. Linear regression between the abundances of cultivable Bacillus and Pseudomonas in bulk soil (A) and rhizosphere soil (B). Fig. S4. Linear regression between the abundances of total and cultivable Bacillus (A, B) and Pseudomonas (C, D) with disease incidence for bulk and rhizosphere soil, respectively. Fig. S5 (A) Biofilm formation of Pseudomonas isolates and B. amyloliquefaciens W19 in monocultures and Pseudomonas-Bacillus cocultures systems for isolates from OF+W19 and OF treatments; (B) Biofilm formation of Pseudomonas isolates and B. amyloliquefaciens W19 in monocultures and Pseudomonas-Bacillus cocultures systems; and (C) Biofilm formation of Pseudomonas isolates and B. amyloliquefaciens W19 in Pseudomonas-Bacillus cocultures systems for isolates from antagonistic group (antagonistic relationship between Pseudomonas spp. and W19) or non-antagonistic group (non-antagonistic relationship between Pseudomonas spp. and W19). Fig. S6. Biofilm formation of Pseudomonas strain PSE78 or PSE82 with B. amyloliquefaciens W19 in monocultures and Pseudomonas-Bacillus co-culture systems (A). Pseudomonas-Bacillus-Fusarium oxysporum f. sp. cubense (FOC) interaction model (B). The interactions between Fusarium oxysporum f. sp. cubense (FOC), B. amyloliquefaciens W19 and Pseudomonas sp. PSE78 or PSE82 (C). Fig. S7. The abundance of total and cultivable F. oxysporum in banana bulk a [file 40168_2020_892_MOESM1_ESM.docx]

**Supplementary Information**

**Supplementary tables**

**Table S1 Sequences of oligonucleotide primers required for quantitative PCR.**

| **Target group** | **Primer name and sequence (5´-3´)** | **Approximate size (bp) of product** | **Annealing temp (℃)** | **Reference** |
| --- | --- | --- | --- | --- |
| Bacteria | Eub338F, ACTCCTACGGGAGGCAGCAG | 200 | 53 | Fierer et al. 2005 |
|  | Eub518R, ATTACCGCGGCTGCTGG |  |  |  |
| Fungi | ITS1f, TCC GTA GGT GAA CCT GCG G | 300 | 53 | Fierer et al. 2005 |
|  | 5.8s, CGC TGC GTT CTT CAT CG |  |  |  |
| *Fusarium oxysporum* | FOF1, ACATACCACTTGTTGCCTCG | 340 | 58 | Jiménez-Fernández et al. 2010 |
|  | FOR1, CGCCAATCAATTTGAGGAACG |  |  |  |
| *Pseudomonas* | Ps-for, GAGTTTGATCCTGGCTCAG | 440 | 50 | Johnsen et al., 1999 |
|  | Ps-rev, CCTTCCTCCCAACTT |  |  |  |
| *Bacillus* | Bs16S1, ATGTTAGCGGCGGACGGGTGAG | 500 | 60 | Mori et al., 2004 |
|  | Bs16SR, AAGTTCCCCAGTTTCCAATGACC |  |  |  |

**Table S2 PERMANOVA results for bacterial and fungal community structure at the OTU level.**

| Soil  compartment | Main test ^a^ | Bacteria | | |  | Fungi | | |
| --- | --- | --- | --- | --- | --- | --- | --- | --- |
|  |  | PERMANOVA | | |  | PERMANOVA | | |
|  |  | F | P (>F) | R^2^ |  | F | *P* (>F) | R^2^ |
| Bulk soil | Treatment | 2.94 | 0.007 | 0.23 |  | 2.06 | 0.106 | 0.17 |
|  | Group | 2.94 | 0.007 | 0.23 |  | 2.18 | 0.093 | 0.18 |
| Rhizosphere soil | Treatments | 2.47 | 0.038 | 0.20 |  | 1.42 | 0.217 | 0.12 |
|  | Group | 2.47 | 0.034 | 0.20 |  | 1.13 | 0.319 | 0.10 |

Note: PERMANOVA, permutational multivariate analysis of variance. ^a^ Main factor represent treatment (OF+W19, SOF+W19, OF, SOF), Group (group1 (OF+W19, SOF+W19), group2 (OF, SOF)). P-values were calculated based on 999 permutations (lowest P-value possible is 0.001). Values in table represent the pseudo-F ratio (F), the level of significance and the estimation of the variance component (R^2^) for PERMANOVA.

**Table S3 Spearman's correlations between Fusarium wilt disease incidence and microbiota determined by Mantel test.**

| microbiota | Compartment | Mantel | |  | PERMANOVA | | |
| --- | --- | --- | --- | --- | --- | --- | --- |
|  |  | R^2^ | P |  | F | P(>F) | R^2^ |
| Bacteria | Bulk | 0.195 | 0.07 |  | 2.489 | 0.016 | 0.199 |
|  | **Rhizosphere** | **0.294** | **0.01** |  | **3.354** | **0.008** | **0.251** |
| Fungi | Bulk | 0.171 | 0.08 |  | 1.593 | 0.194 | 0.137 |
|  | Rhizosphere | 0.005 | 0.42 |  | 1.168 | 0.307 | 0.105 |

Note: PERMANOVA, permutational multivariate analysis of variance. p-values were calculated based on 999 permutations (lowest P-value possible is 0.001). Values in table represent the pseudo-F ratio (F), the level of significance and the estimation of the variance component (R^2^) for PERMANOVA.

**Supplementary Figures**

**Fig. S1. Linear regression between the abundances of cultivable and total *F*. *oxysporum* and relative abundance of *Fusarium* with disease incidence for bulk (A) and rhizosphere (B) soil.**


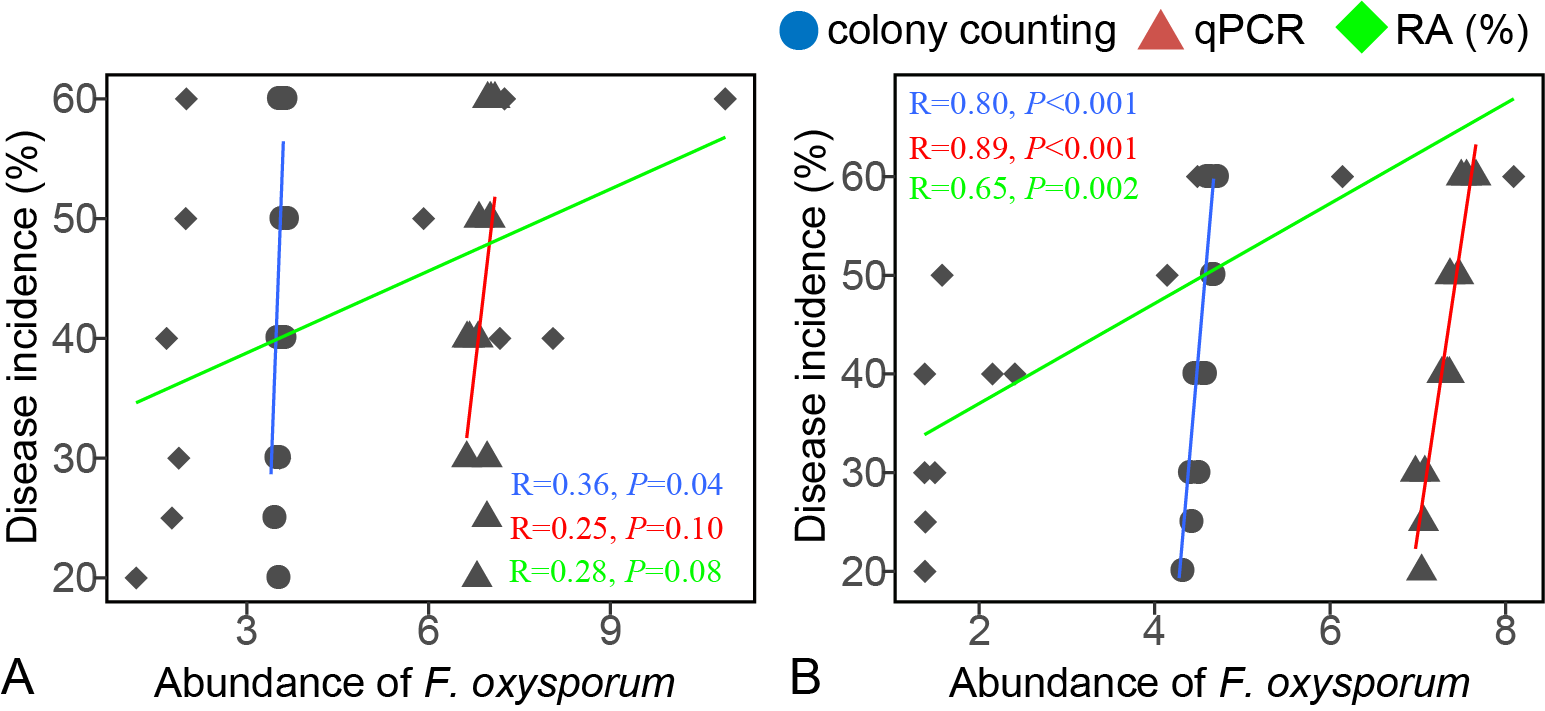


Note: Colony counting (lg CFU per gram soil), qPCR (lg copies per gram soil) and relative abundance (RA (%)).

**Fig. S2. The abundance of total and cultivable *Bacillus* (A) and *Pseudomonas* (B) in banana bulk and rhizosphere soil.**


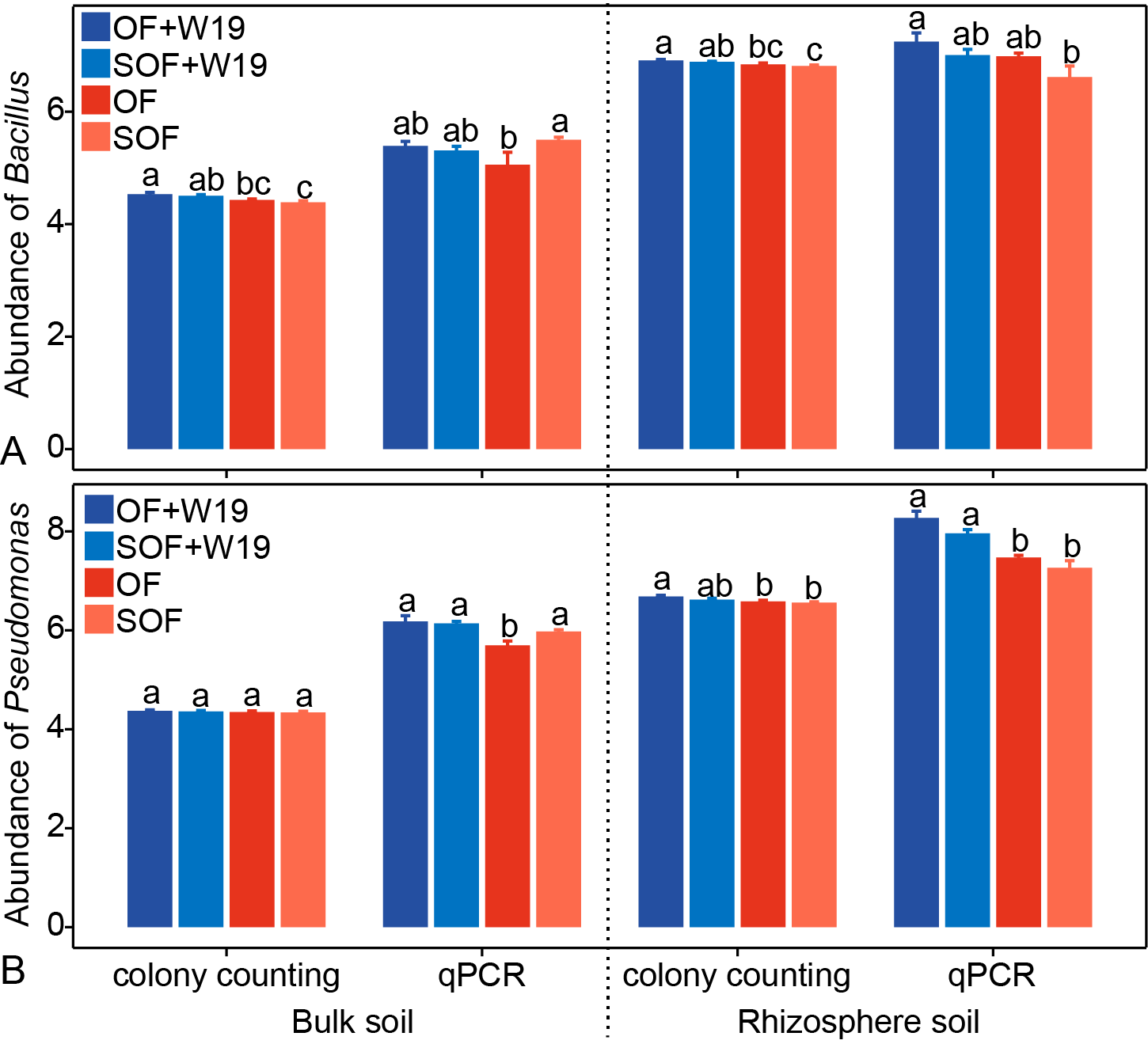


OF+W19 = Bio-organic fertilizer containing *B. amyloliquefaciens* W19, SOF + W19 = sterilized bio-organic fertilizer inoculated with *B*. *amyloliquefaciens* W19, OF = Organic fertilizer, SOF = Sterilized organic fertilizer. Different letters above the bars indicate a significant difference at the 0.05 probability level according to the Duncan test. Colony counting (lg CFU per gram soil), qPCR (lg copies per gram soil).

**Fig. S3. Linear regression between the abundances of cultivable *Bacillus* and *Pseudomonas* in bulk soil (A) and rhizosphere soil (B).**


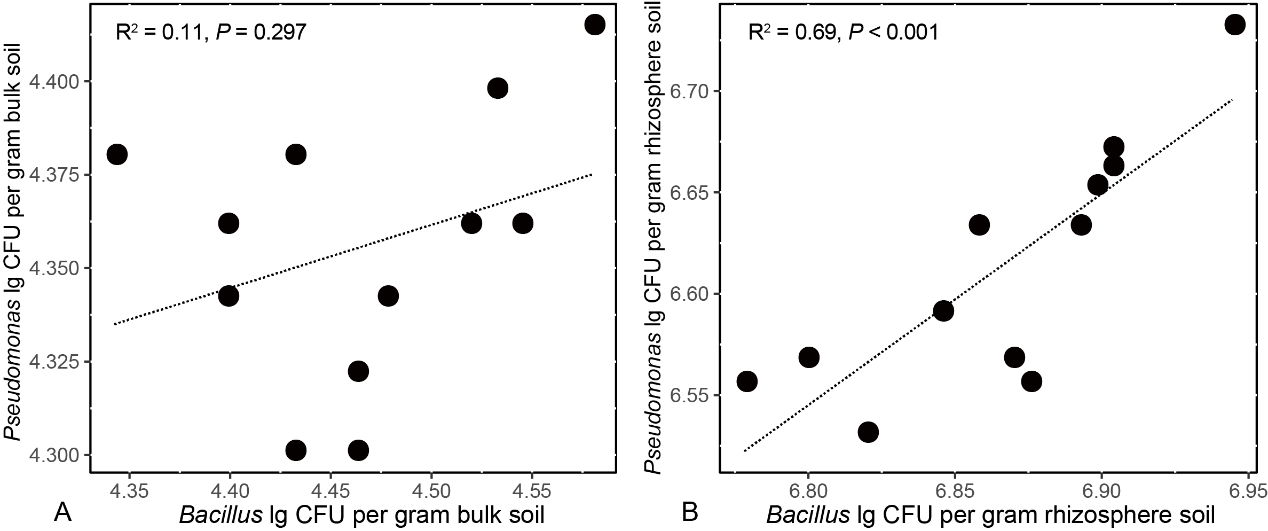


**Fig. S4. Linear regression between the abundances of total and cultivable *Bacillus* (A, B) and *Pseudomonas* (C, D) with disease incidence for bulk and rhizosphere soil, respectively.**


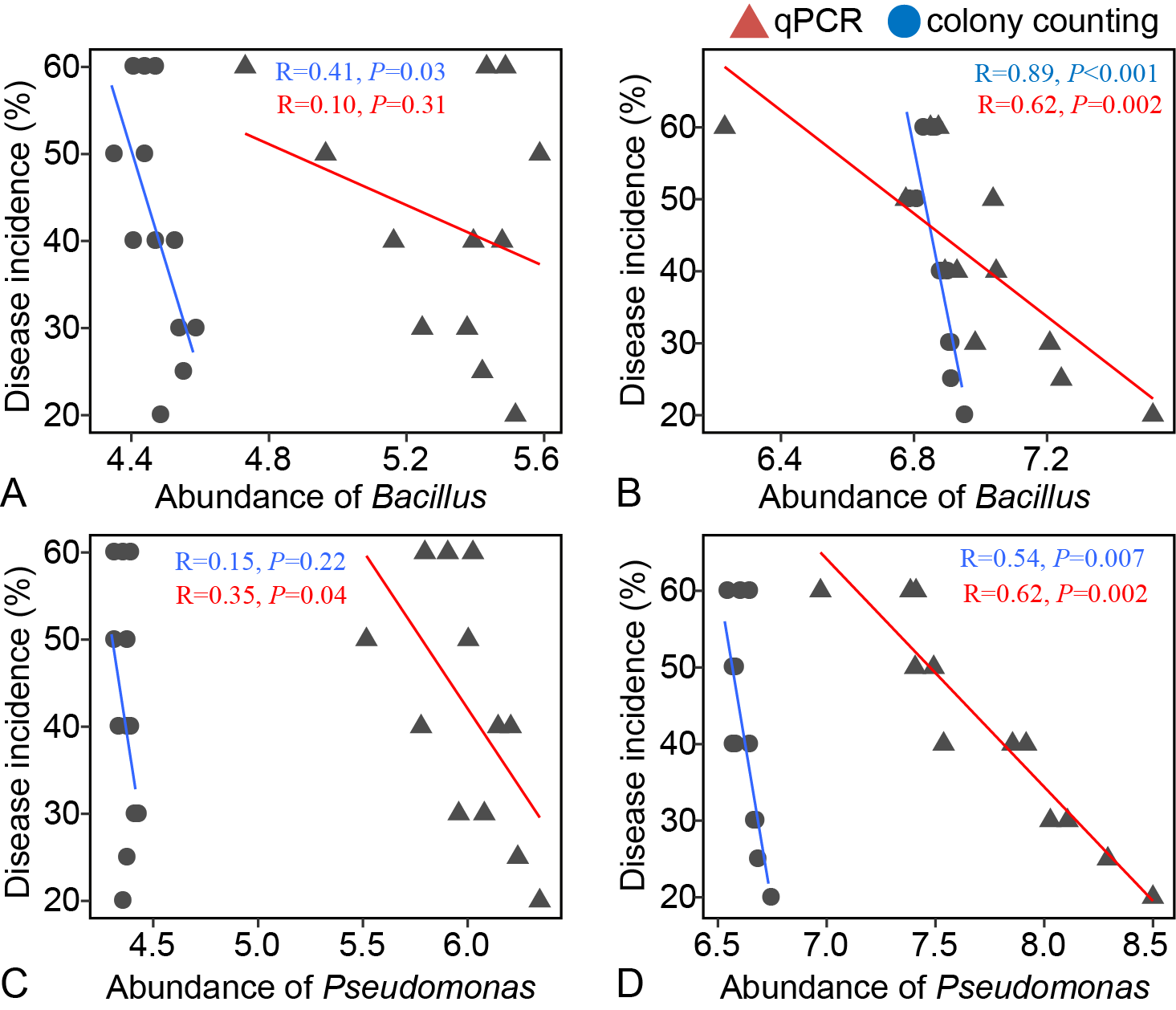


Note: Colony counting (lg CFU per gram soil), qPCR (lg copies per gram soil).

**Fig. S5 (A) Biofilm formation of *Pseudomonas* isolates and *B. amyloliquefaciens* W19 in monocultures and *Pseudomonas-Bacillus* cocultures systems for isolates from OF+W19 and OF treatments; (B) Biofilm formation of *Pseudomonas* isolates and *B. amyloliquefaciens* W19 in monocultures and *Pseudomonas-Bacillus* cocultures systems; and (C) Biofilm formation of *Pseudomonas* isolates and *B. amyloliquefaciens* W19 in *Pseudomonas-Bacillus* cocultures systems for isolates from antagonistic group (antagonistic relationship between *Pseudomonas* spp. and W19) or non-antagonistic group (non-antagonistic relationship between *Pseudomonas* spp. and W19).**


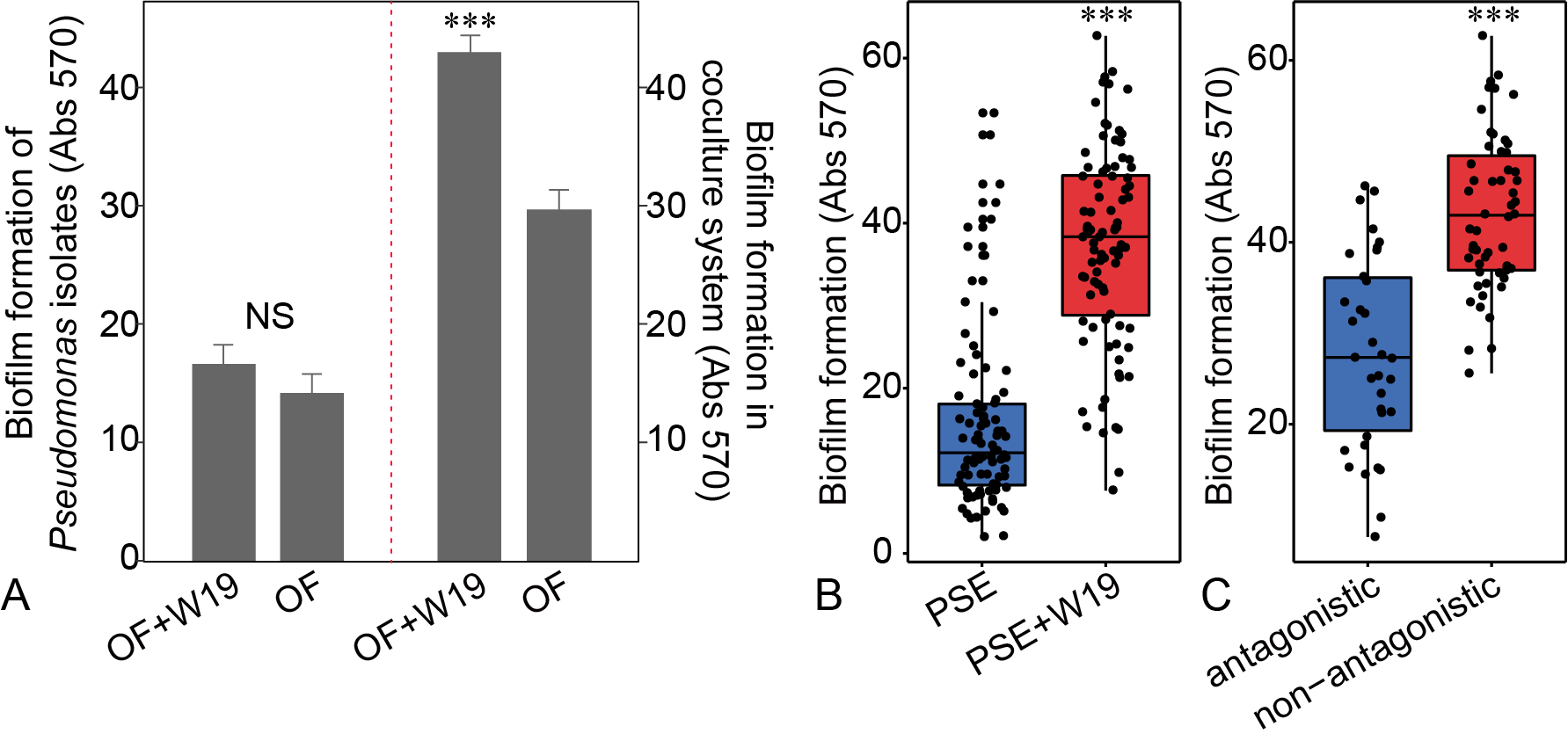


OF+W19 = Bio-organic fertilizer containing *B. amyloliquefaciens* W19, OF = Organic fertilizer. Wilcoxon-test, ****P* < 0.001.

**Fig. S6. Biofilm formation of *Pseudomonas* strain PSE78 or PSE82 with *B. amyloliquefaciens* W19 in monocultures and *Pseudomonas-Bacillus* co-culture systems (A). *Pseudomonas*-*Bacillus*-*Fusarium oxysporum* f. sp. *cubense* (FOC) interaction model (B). The interactions between *Fusarium oxysporum* f. sp. *cubense* (FOC), *B*. *amyloliquefaciens* W19 and *Pseudomonas* sp. PSE78 or PSE82 (C).**

**
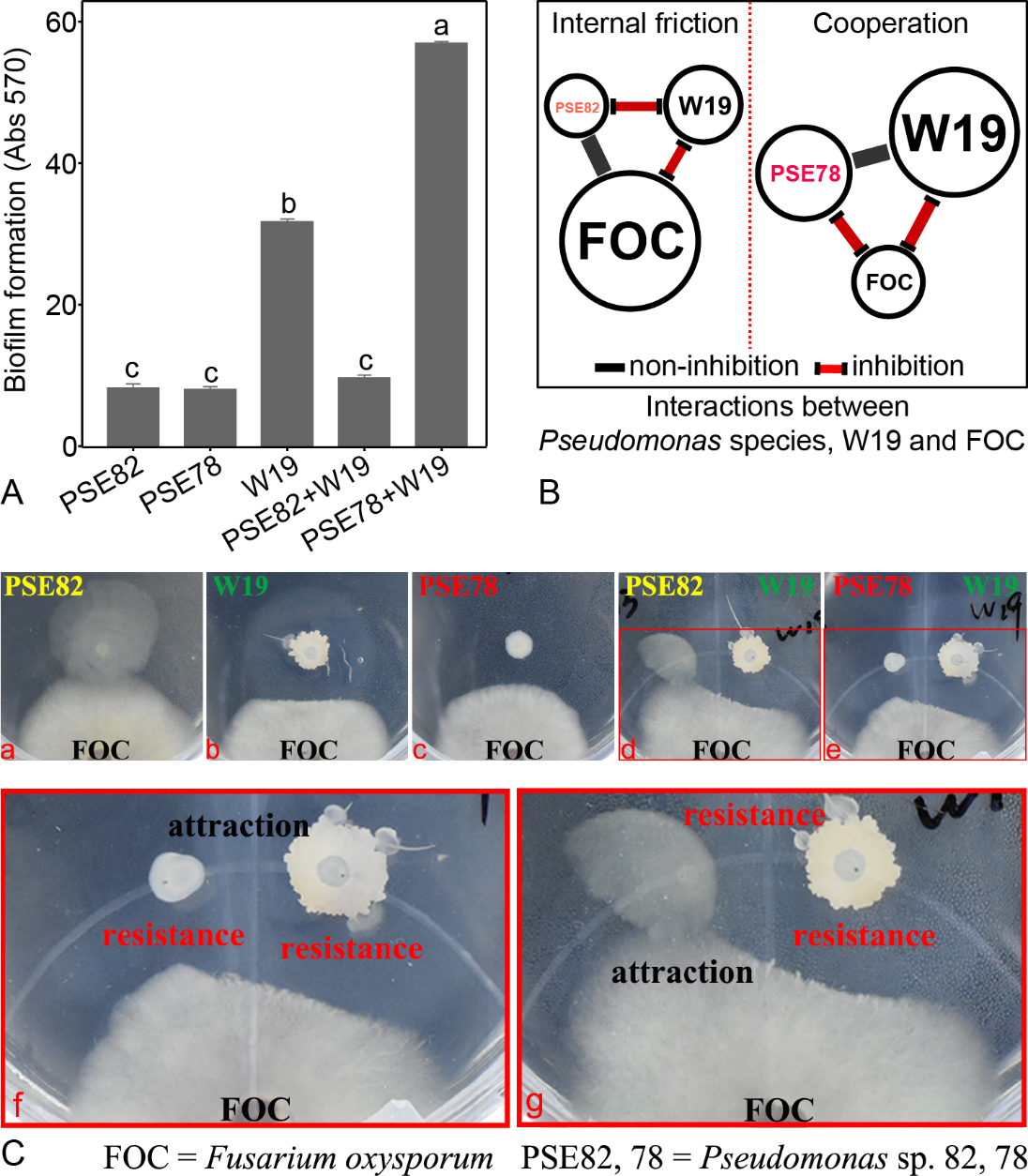
**

Note: Different letters indicate a significant difference at the 0.05 probability level according to the Duncan test.

**Fig. S7. The abundance of total and cultivable *F. oxysporum* in banana bulk and rhizosphere soil treated with sterilized organic fertilizer inoculated with either *Pseudomonas* PSE78 or PSE82 as compared to sterilized organic fertilizer (SOF) and chemical fertilizer (CK) treatments.**

**
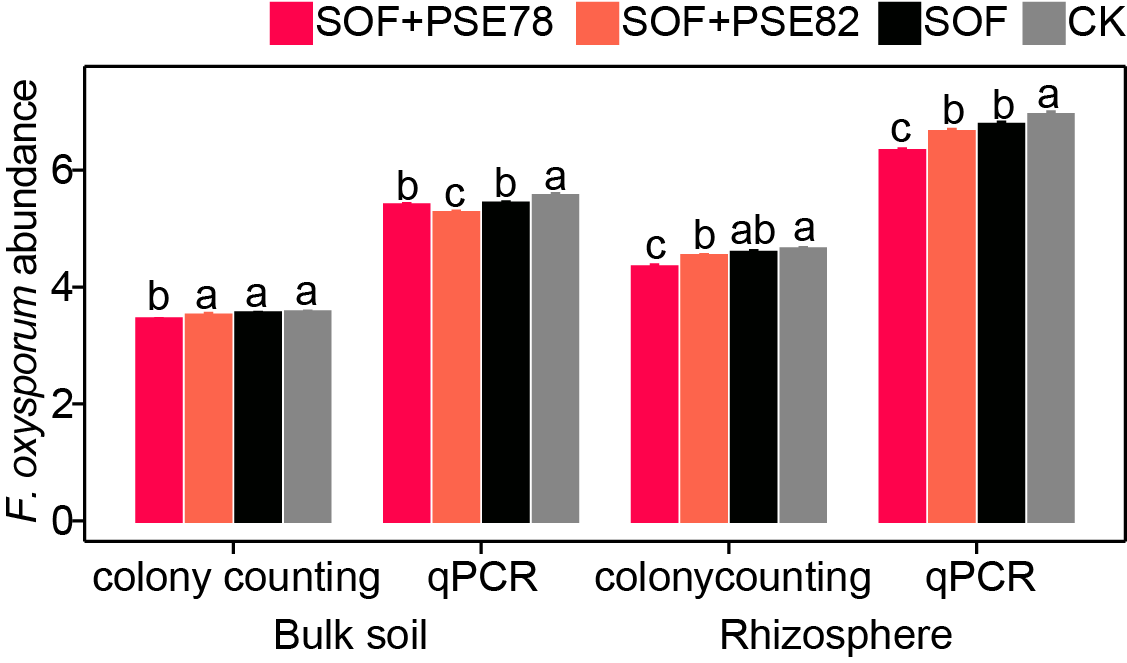
**

Note: Different letters above the bars indicate a significant difference at the 0.05 probability level according to the Duncan test. Colony counting (lg CFU per gram soil), qPCR (lg copies per gram soil).

**Fig. S8. The abundance of total and cultivable *Pseudomonas* and *Bacillus* in banana bulk and rhizosphere soil treated with sterilized organic fertilizer inoculated with either *Pseudomonas* PSE78 or PSE82 as compared to sterilized organic fertilizer (SOF) and chemical fertilizer (CK) treatments.**

**
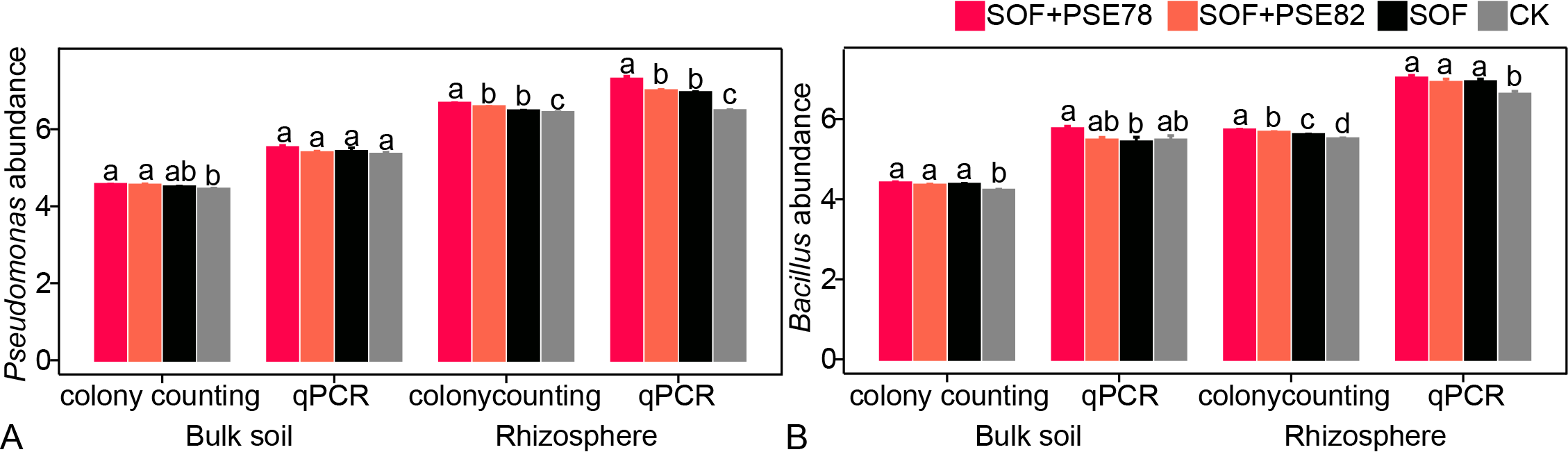
**

Note: Different letters above the bars indicate a significant difference at the 0.05 probability level according to the Duncan test. Colony counting (lg CFU per gram soil), qPCR (lg copies per gram soil).

**Fig. S9. Linear regression between total and cultivable *Fusarium*, *Pseudomonas* and *Bacillus* with disease incidence in bulk soil (A, B, and C) and rhizosphere soil (E, F, and G). Linear regression between total and cultivable *Bacillus* and *Pseudomonas* in bulk soil (D) and rhizosphere soil (H).**

**
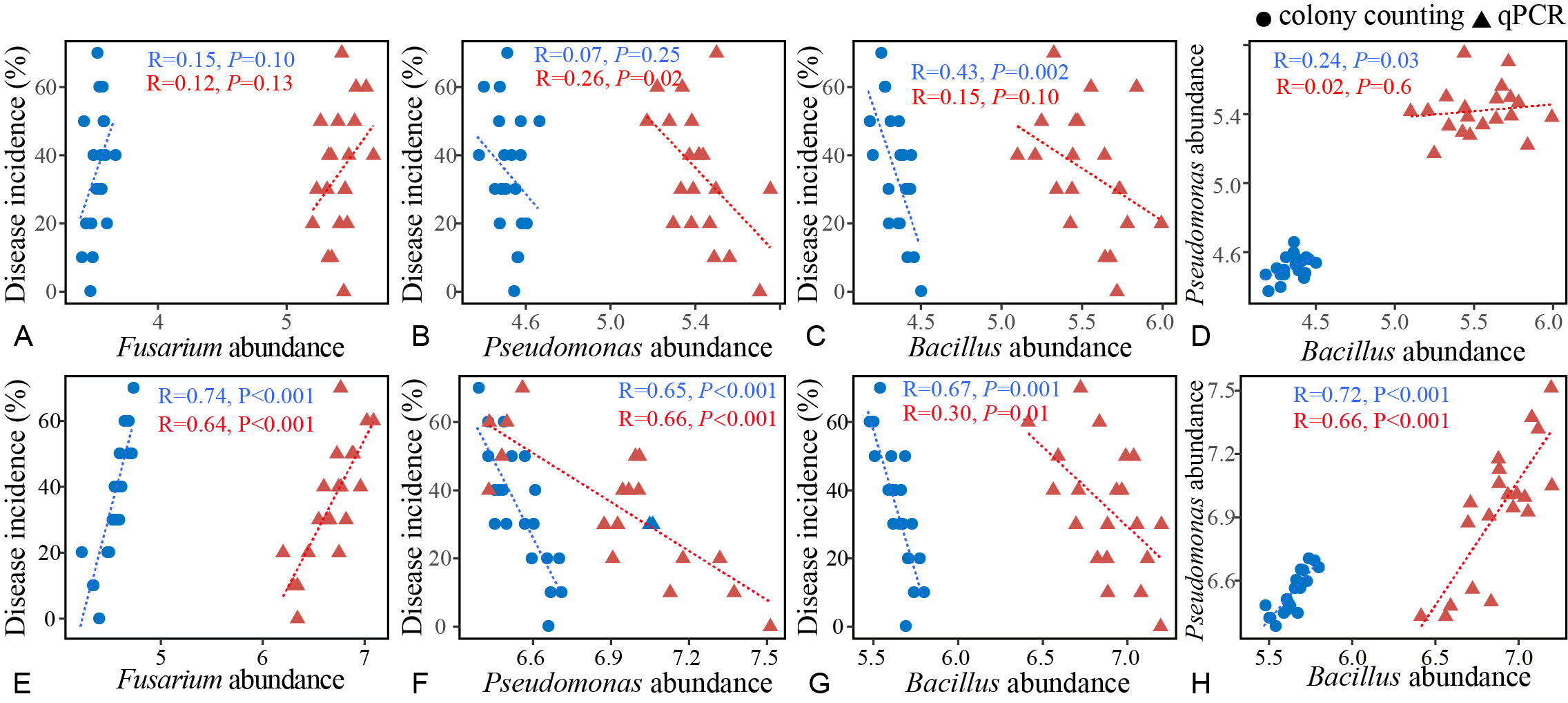
**

Note: Colony counting (lg CFU per gram soil), qPCR (lg copies per gram soil).

**Fig. S10. Schematic representation of the experimental design for testing combined impacts of PSE strains and *Bacillus amyloliquefaciens* W19 on plant disease. (I) Sterile banana seedlings were inoculated with *Bacillus amyloliquefaciens* W19, *Pseudomonas* sp. PSE78 (PSE78), or *Pseudomonas* sp. PSE82 (PSE82), or a combination of W19 and PSE78 (W19+PSE78) or W19 and PSE82 (W19+PSE82) or mock treated (Control). (II) Thirty days after inoculation, all plants were transplanted into a new sterile substrate, (III) after which the banana plants were either inoculated with *Fusarium oxysporum* f. sp. *cubense* (FOC) spore suspension or given a mock inoculation. Disease severity was quantified by counting the number of FOC that colonized on plant roots after 3 weeks of inoculation.**


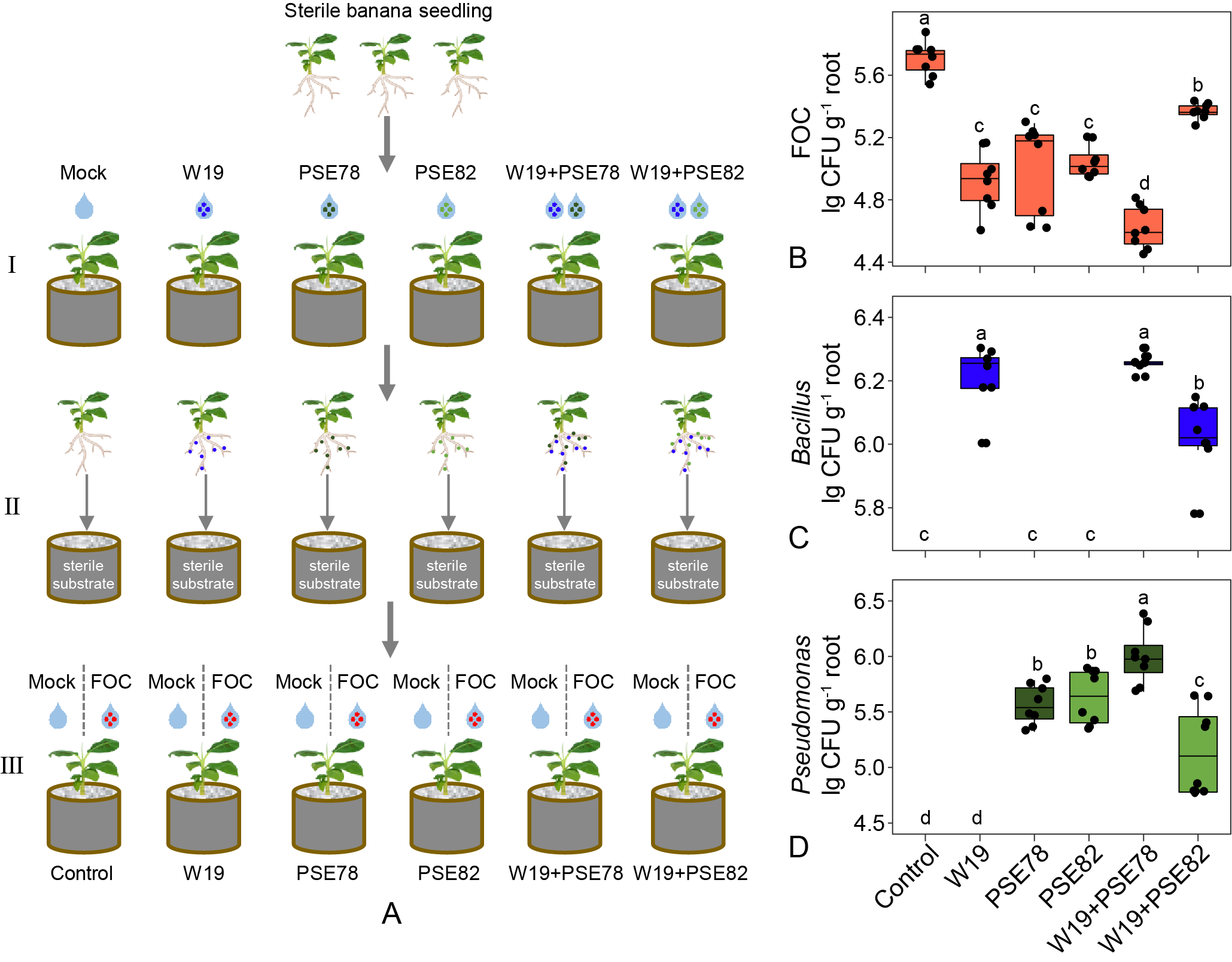


**Supplementary results**

**Illumina Miseq sequencing data description**

We sequenced the V4 and ITS1 regions of the 16S rRNA and ITS genes for 24 samples, which yielded 2542433 and 1076377 high-quality reads for subsequent analysis, respectively. The number of retained bacterial 16S rRNA gene sequences per sample varied from 79617 to 177893 with an average of 105935 sequences, and a range of 27890 to 100570 with an average of 44849 sequences per sample for fungi was obtained. The rarefaction curves of all soil samples based on the OTU numbers are shown in Fig. S11. After discarding low-abundance and non-bacterial and fungal operational taxonomic units (OTUs), we obtained 3797 bacterial and 1097 fungal OTUs. 99.7% of the bacterial sequences could be assigned to 25 bacterial phyla, mainly including Proteobacteria, Bacteroidetes, Acidobacteria, Actinobacteria and Gemmatimonadetes (Fig. S12A); and 86.8% of the fungal sequences could be assigned to 5 fungal phyla, including Ascomycota, Basidiomycota, Chytridiomycota, Glomeromycota and Zygomycota (Fig. S12B). In total, 691 bacterial and 178 fungal genera belonging to 206 bacterial and 92 fungal families were recorded from these samples, respectively.


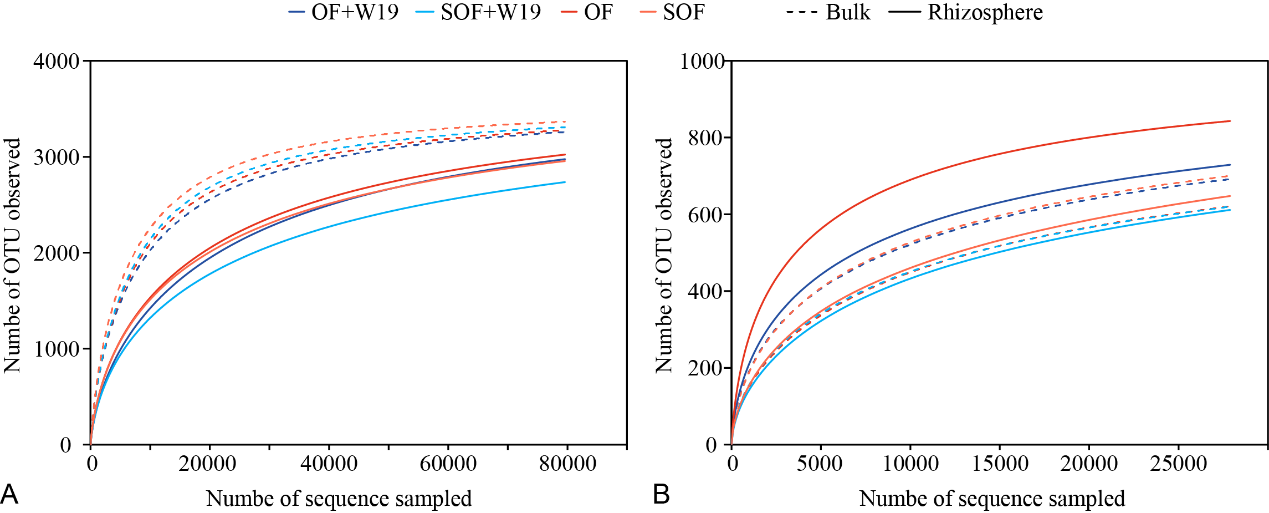


Fig. S11. Rarefaction curves of 16S rRNA genes (bacteria) and ITS sequences (fungi) at 97 % similarity levels of the bulk and rhizosphere soil. OF+W19 = Bio-organic fertilizer containing *B*. *amyloliquefaciens* W19, SOF+W19 = sterilized bio-organic fertilizer inoculated with *B*. *amyloliquefaciens* W19, OF = Organic fertilizer, SOF = Sterilized organic fertilizer.


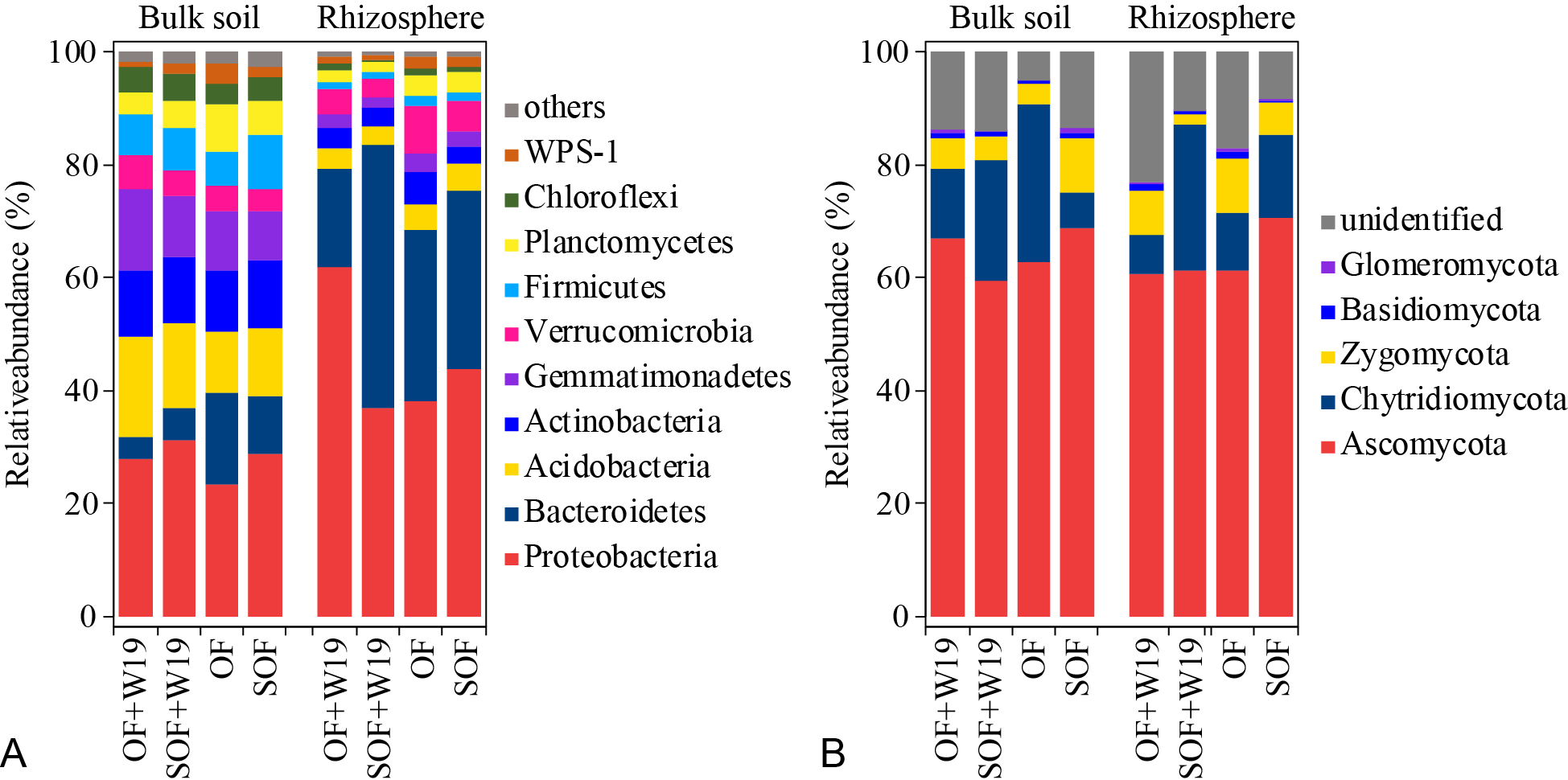


Fig. S12. The relative abundance of bacterial (dominate bacterial phyla) and fungal phyla in bulk and banana rhizosphere soils. OF+W19 = Bio-organic fertilizer containing *B*. *amyloliquefaciens* W19, SOF+W19 = sterilized bio-organic fertilizer inoculated with *B*. *amyloliquefaciens* W19, OF = Organic fertilizer, SOF = Sterilized organic fertilizer.

**Microbial community *alpha* diversity**

Microbial community richness and diversity were determined using the randomly re-sampled 79617 bacterial and 27890 fungal sequences based on the Sobs richness and the Shannon diversity (H ) index (Fig. S13).

In bulk soil, significantly (*P* < 0.05) higher bacterial richness and diversity were observed in SOF treatment as compared to other treatments, while OF+W19 and SOF treatments both significantly (*P* < 0.05) increased the fungal richness as compared to SOF+W19 and OF treatments. In addition, significantly (*P* < 0.05) higher fungal diversity was observed in OF+W19 and SOF treatments as compared to OF treatment.

In banana rhizosphere soil, significantly (*P* < 0.05) higher bacterial richness was observed in OF treatment as compared to other treatments. In addition, OF and SOF treatments both significantly (*P* < 0.05) increased the bacterial diversity as compared to OF+W19 treatment. For fungi, OF+W19 and OF treatments both significantly (*P* < 0.05) increased the fungal richness and diversity as compared to SOF+W19 and SOF treatments, while highest fungal richness and diversity were observed in OF treatment.


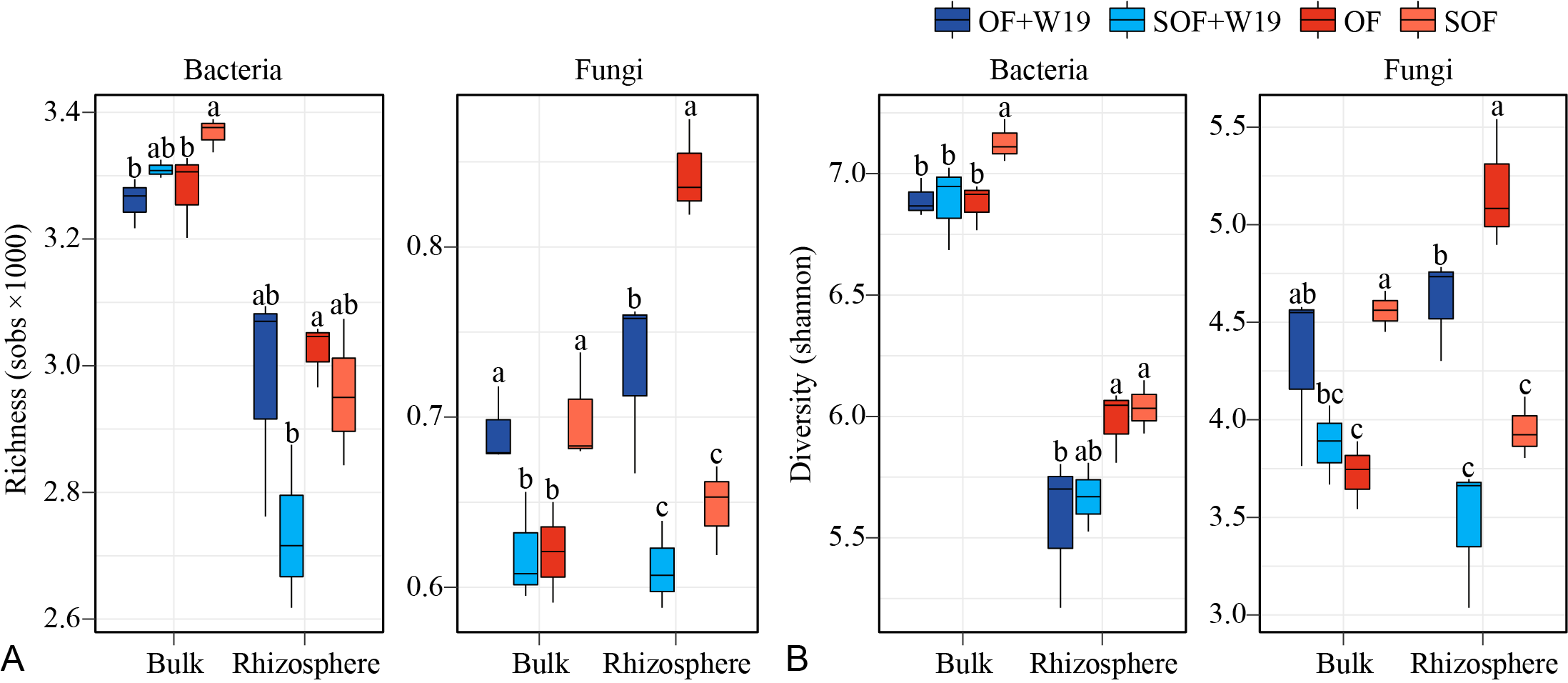


Fig. S13. Bacterial and fungal richness (Sobs) (A) and diversity (Shannon) (B) in bulk and banana rhizosphere soils. Different letters indicate significant difference at the 0.05 probability level according to the Duncan test. OF+W19 = Bio-organic fertilizer containing *B*. *amyloliquefaciens* W19, SOF+W19 = sterilized bio-organic fertilizer inoculated with *B*. *amyloliquefaciens* W19, OF = Organic fertilizer, SOF = Sterilized organic fertilizer.
